# A hard molecular nanomagnet from confined paramagnetic 3d-4f spins inside a fullerene cage

**DOI:** 10.1038/s41467-023-44194-y

**Published:** 2023-12-19

**Authors:** Chenli Huang, Rong Sun, Lipiao Bao, Xinyue Tian, Changwang Pan, Mengyang Li, Wangqiang Shen, Kun Guo, Bingwu Wang, Xing Lu, Song Gao

**Affiliations:** 1 State Key Laboratory of Materials Processing and Die & Mould Technology, School of Materials Science and Engineering, Huazhong University of Science and Technology, 1037, Luoyu Road, Wuhan, 430074 P. R. China; 2 Beijing National Laboratory of Molecular Science, State Key Laboratory of Rare Earth Materials Chemistry and Applications, College of Chemistry and Molecular Engineering, Peking University, Beijing, 100871 P. R. China; 3 School of Physics, Xidian University, Xi’an, 710071 China; 4 College of Chemistry and Chemical Engineering, Hainan University, No. 58, Renmin Avenue, Haikou, 570228 P. R. China; 5 Spin-X Institute, School of Chemistry and Chemical Engineering, State Key Laboratory of Luminescent Materials and Devices, Guangdong-Hong Kong-Macao Joint Laboratory of Optoelectronic and Magnetic Functional Materials, South China University of Technology, Guangzhou, 510641 P. R. China

**Keywords:** Organometallic chemistry, Organometallic chemistry, Carbon nanotubes and fullerenes, Magnetic devices, Magnetic properties and materials

## Abstract

Reducing inter-spin distance can enhance magnetic interactions and allow for the realization of outstanding magnetic properties. However, achieving reduced distances is technically challenging. Here, we construct a 3d-4f metal cluster (Dy_2_VN) inside a C_80_ cage, affording a heretofore unseen metallofullerene containing both paramagnetic 3d and 4f metal ions. The significantly suppressed 3d-4f (Dy-V) distances, due to the unique cage confinement effect, were observed by crystallographic and theoretical analysis of Dy_2_VN@*I*_*h*_(7)-C_80_. These reduced distances result in an enhanced magnetic coupling (*J*_total, Dy-V_ = 53.30 cm^−1^; *J*_total, Dy-Dy_ = −6.25 cm^−1^), leading to a high magnetic blocking temperature compared to reported 3d-4f single-molecule magnets and strong coercive field of 2.73 Tesla. Our work presents a new class of single-molecule magnets with both paramagnetic 3d and 4f metals confined in a fullerene cage, offering superior and tunable magnetic properties due to the unique cage confinement effect and the diverse composition of the entrapped magnetic core.

## Abstract (web summary)

Shortening the inter-spin distance is an effective way to enhance magnetic coupling. However, it is typically challenging to change the inter-ion distance in most magnetic systems. Here, Huang et al present a strategy for enhancing magnetic interactions, by confining a molecular magnetic system inside a carbon fullerene cage, leading to enhanced magnetic properties.

## Introduction

Magnets are omnipresent in daily life and play a prominent role in modern materials research and industry^1–3^, spanning from magneto-mechanical machines to spintronics, high-density information storage and so on^4–8^. Interactions between magnetic moment carriers are crucial to the construction of magnets^9^. The search for the origins and roles of magnetic interactions has shaped the development of not only magnets^10–13^ but also magnetic semiconductors^14,15^, and even superconductors^16^.

Spin centers magnetically couple with each other in two ways, the dipolar interaction and the exchange interaction^17,18^. The dipolar interaction arises from the influence of the magnetic field generated by one of the spin centers on the other and is distance-dependent^17^, approximately inversely correlated to the cube of their distance. The essence of the exchange interaction is the overlap of magnetic orbitals or spin densities^18,19^. Strong magnetic interactions are attractive due to the resulting magnetic properties. As a well-known example, strong interactions between the itinerant electrons in 3d transition metals and the 5d conduction band plus localized 4f electrons in lanthanides result in large magnetocrystalline anisotropies and high Curie temperature in rare earth-cobalt-based permanents, in particular making SmCo_5_ the first commercial rare-earth permanent magnets^10,11^.

Unfortunately, obtaining strong magnetic interactions in molecular systems is challenging. The large ionic radius and shielding 4 f orbits of lanthanide ions lead to weak 4f-4f interactions (usually smaller than 1 cm^−1^)^20,21^ and weak 3d-4f interactions (usually smaller than 10 cm^−1^)^22–24^. While shortening inter-spin distances could be an effective approach for enhancing magnetic coupling, metal spin centers in conventional coordination chemistry tend to be stabilized through definite coordination saturation, rendering effective distance reduction difficult due to the unavoidable solvent and ligand effects^25^. Nevertheless, the above mentioned strong magnetic interactions in molecular systems play an important role, for an especially relevant example here, to help design high-performance single-molecule magnets (SMMs)—which may exhibit slow magnetic relaxation owing to their intrinsic anisotropy^26^.

One promising and potentially straightforward approach for achieving strong magnetic interactions is to confine multiple magnetic metal centers within a fullerene cage thereby exploiting the unique coordination environment within the fullerene cage^27–32^. The spatial confinement effect of fullerenes can effectively reduce the distances between spin centers and enhance the magnetic interaction. In addition, the shielding effect of the cages could also stabilize the internal clusters and make endohedral-metallofullerenes (EMFs) air-stable, essential for prospective applications. However, simultaneously introducing 3d and 4f spin centers into fullerene cages has never been reported due to the distinct synthetic difficulty.

Herein, we explored such class of metallofullerene containing both paramagnetic 4f and 3d metal elements, taking Dy_2_VN@*I*_*h*_(7)-C_80_ as an example, whose molecular structure was unambiguously determined by X-ray crystallography. Due to the unique confinement effect of the fullerene cage on the endohedral Dy_2_VN cluster, the distances between the spin centers are significantly suppressed. The resulting enhanced magnetic interactions between confined spin centers (*J*_total, Dy-V_ = 53.30 cm^−1^; *J*_total_, _Dy-Dy_ = −6.25 cm^−1^) inhibit quantum tunneling of magnetization (QTM), which is the main factor hindering the performance of reported SMMs, and lead to a high blocking temperature (*T*_B_) for 3d-4f SMMs and huge coercive field (*H*_c_).

## Results

### Synthesis and crystal structure of Dy_2_VN@*I*_*h*_(7)-C_80_

**Fig. 1:**
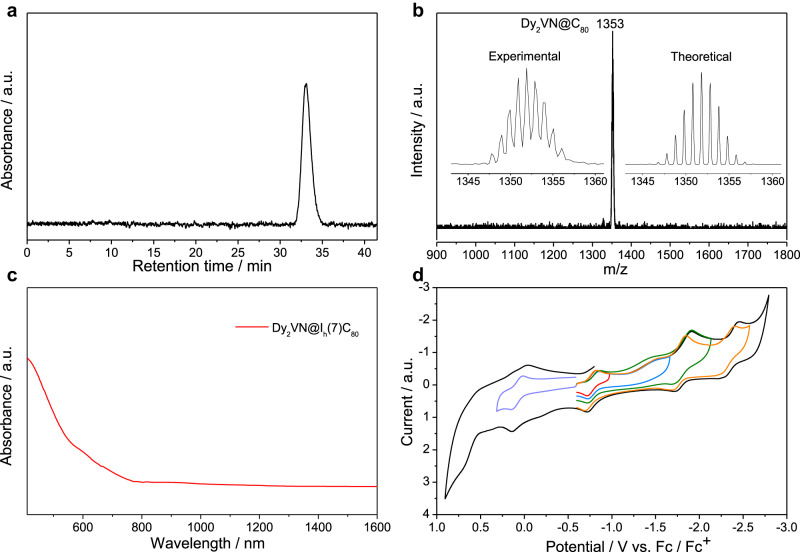
Characterizations of Dy_2_VN@*I*_*h*_(7)-C_80_. **a** HPLC profile, **b** high-resolution mass spectrum, **c** absorption spectrum, and **d** CV curves of Dy_2_VN@*I*_*h*_(7)-C_80_. Electrochemical conditions: 0.05 M tetrabutylammonium hexafluorophosphate (TBAPF_6_) in o-dichlorobenzene (*o-*DCB) as electrolyte and 100 mV mL s^-1^ scan rate.

Dy_2_VN@*I*_*h*_(7)-C_80_ was synthesized via a direct arc-discharge method and purified by high-performance liquid chromatographic (HPLC) separation. The analytical HPLC profile and high-resolution mass spectrum of Dy_2_VN@*I*_*h*_(7)-C_80_ are shown in Fig. 1. The electronic properties of Dy_2_VN@*I*_*h*_(7)-C_80_ were investigated by vis-NIR spectroscopic and cyclic voltametric (CV) studies. The absorption spectrum (Fig. 1c) of Dy_2_VN@*I*_*h*_(7)-C_80_ displays four absorption peaks at 465, 581, 663, and 773 nm with the absorption onset at ~1424 nm, corresponding to an optical bandgap of 0.87 eV. These absorption bands have significant red shifts compared to those of the reported Dy_2_ScN@*I*_*h*_(7)-C_80_ (absorption bands: 406, 565, 676, and 705 nm)^33^, suggesting the crucial role of the entrapped V atom on the electronic structure of the whole EMF molecule. The recorded CV curves are shown in Fig. 1d and the characteristic redox potentials are listed in Table S4. Dy_2_VN@*I*_*h*_(7)-C_80_ has one reversible oxidation process (0.05 V) and four reduction processes (-0.81, -1.53, -1.81, and -2.35 V), in which the first, third and fourth reduction processes are all reversible, but the second reduction process is irreversible. The first oxidation potential and the first reduction potential are 0.05 V and -0.81 V, respectively, resulting in an electrochemical bandgap of 0.86 eV, which is in perfect agreement with its optical bandgap.

**Fig. 2:**
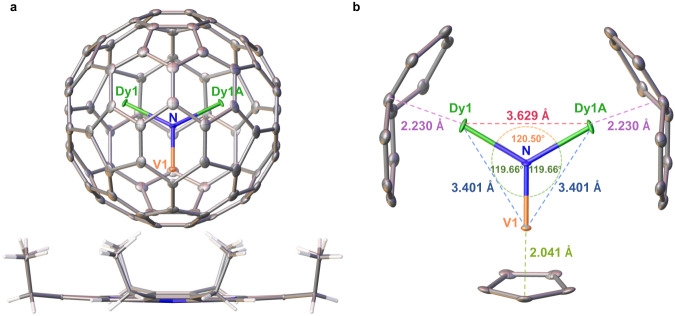
Crystal structure of Dy_2_VN@*I*_*h*_(7)-C_80_. **a** Ortep drawing of Dy_2_VN@*I*_*h*_(7)-C_80_ co-crystallized with Ni(OEP), where the thermal ellipsoids are set at 10% probability. Only one orientation of the fullerene cage together with the major site of Dy_2_VN cluster is shown and solvent molecules are omitted for clarity. **b** The positions of the major Dy_2_VN site with respect to the nearest carbon atoms of the *I*_*h*_(7)-C_80_ cage. Green: Dy; Blue: N; Orange: V; Gray: C.

The molecular structure of Dy_2_VN@*I*_*h*_(7)-C_80_ and interactions of the mixed metal atoms inside the cage are studied by X-ray crystallography (Fig. 2). Within the cage, the N atom is fully ordered, which locates on the crystallographic symmetric plane. Albeit of some disorder for Dy and V (Fig. S2 and Table S2), the major sites can be clearly distinguished. Dy1 with an occupancy value of 0.43 is the major Dy site and Dy1A generated by crystallographic operations from Dy1 is the other. The major V site locates on the crystallographic mirror plane with the occupancy value of 0.44. Due to the much smaller ion radius of V (0.64 Å) than that of Dy (0.91 Å), their metal-cage and metal-N distance show remarkable differences. The distance between Dy1 or Dy1A and the nearest cage carbon atom is 2.230 Å, whereas the distance between V1 and the nearest cage carbon atom is 2.041 Å. The relative orientation between the inner metals and the cage resembles that of Sc_2_VN@*I*_*h*_(7)-C_80_ which also contains the transition metal V^34^, but is obviously different from those of the reported lanthanide metal-based EMFs, such as MSc_2_N@*I*_*h*_(7)-C_80_ (M=La, Ce, Gd, Tb)^35–37^ where the large lanthanide metal atom resides under the centers of hexagons while the small Sc atoms are close to an intersection between a hexagon and a pentagon. This dramatic difference demonstrates the crucial role of the entrapped transition metal V in the metal-cage interactions.

Within the Dy_2_VN cluster, the bond length of Dy1-N is 2.090 Å while the V-N bond is significantly shorter (1.841 Å). These distinct differences allow us to unambiguously identify V from Dy, which is difficult to distinguish in other metallofullerenes containing mixed metals such as Sc_3-x_V_x_N@C_80_(x = 1–2)^34,38^. The included angles of Dy1-N-V1 and Dy1-N-Dy2 are 119.66° and 120.50°, respectively, so that the sum of the three angles is 359.82° (≈360°), indicative of a planar structure of the Dy_2_VN cluster. With the spatial confinement effect of the fullerene cage on the endohedral Dy_2_VN cluster, the Dy-Dy distance has been significantly suppressed to 3.629 Å, which is the shortest among all the reported values for 3d-4f SMMs, as summarized in Table 1. The strong cage confinement is further reflected by the ultrashort Dy-V distance of 3.401 Å, which represents one of the smallest 3d-4f distances among all the reported 3d-4f SMMs. The compressed nature of the Dy_2_VN cluster inside the cage is further verified by DFT calculations (Figure S7). The optimized geometry of Dy_2_VN@*I*_*h*_(7)-C_80_ resembles the crystal structure and in particularly confirms the ultrashort metal-metal distances. The calculated Dy-Dy and Dy-V distances are 3.631 Å and 3.491/3.331 Å, respectively, which are comparable to the recorded values in crystal structures (*vide supra*). Additional theoretical analysis shows weak Dy-Dy and Dy-V bonding with respective Wiberg bond order of 0.05 and 0.12/0.15, respectively. Such a unique cage confinement effect on the metals strengthens the interactions between the anisotropic lanthanide metals, and enhances the 3d-4f magnetic coupling, slowing down the magnetic relaxation and improving the SMM performance (*vide infra*).

**Table 1:** Dy-Dy and 3d-4f distances in representative 3d-4f SMMs

3d-4f SMMs	Dy-Dy distance / Å	3d-4f distance / Å	ref.
Dy_2_V^III^N	3.629	3.401	*this work*
DyCu^II^_5_	N/A	3.904 ~ 3.964	^39^
Dy_2_Cu^II^_10_	4.291	3.899 ~ 3.965	^39^
Dy_2_Cr^III^_2_	4.105	3.290 ~ 3.302	^40^
Dy_2_Fe^III^_4_	3.952	3.431 ~ 3.450 / 5.766 ~ 5.973	^41^
DyFe^II^_2_	N/A	3.520 ~ 3.599	^22^
Dy_2_Co^II^_2_	6.183	3.489 ~ 3.496	^23^
Dy_2_Mn^II^_2_	6.292	3.553 ~ 3.613	^24^
DyV^IV^O	N/A	3.474	^42^
DyFe^0^	N/A	2.88	^43^

Dy_2_V^III^N = Dy_2_V^III^N@C_80_; DyCu^II^_5_ = [DyCu^II^_5_(quinha)_5_(sal)_2_(py)_5_]-(CF_3_SO_3_)·py·4H_2_O;^39^

Dy_2_Cu^II^_10_ = [Dy_2_Cu^II^_10_(quinha)_10_ (sal)_2_(OH)(py)_9_] (CF_3_SO_3_)_3_·2py·2CH_3_OH· 2H_2_O;^39^

Dy_2_Cr^III^_2_ = Cr^III^_2_Dy_2_(OMe)_2_(O_2_CPh)_4_(mdea)_2_(NO_3_)_2_;^40^

Dy_2_Fe^III^_4_ = [Fe^III^_4_Dy_2_(*μ*_3_-OH)_2_ (mdea)_6_(SCN)_2_(NO_3_)_2_(H_2_O)_2_]·4H_2_O·2MeCN;^41^

DyFe^II^_2_ = [Fe^II^_2_Dy(L)_2_(H_2_O)]ClO_4_·2H_2_O, L = 2,2′,2′′-(((nitrilotris(ethane-2,1-diyl))tris(azanediyl))tris(methylene))tris(4-chlorophenol);^22^

Dy_2_Co^II^_2_ = [Co^II^_2_Dy_2_(L)_4_(NO_3_)_2_(THF)_2_] ·4THF, H_2_L = (*E*)-2-(2-hydroxy-3-methoxybenzylideneamino)phenol;^23^ Dy_2_Mn^II^_2_ = Dy_2_Mn^II^_2_(L)_4_(NO_3_)_2_(DMF)_2_, H_2_L = (*E*)-2-ethoxy-6-(((2-hydroxyphenyl)imino)methyl)phenol;^24^

DyV^IV^O = Dy(V^IV^O)L(NO_3_)_3_(H_2_O), H_2_L = N, N′-bis(1-hydroxy-2-benzylidene-6-methoxy)−1,7-diamino-4-azaheptane;^42^

DyFe^0^ = PyCp_2_Dy-FeCp(CO)_2_, PyCp_2_^2− ^= [2,6-(CH_2_C_5_H_3_)_2_C_5_H_3_N]^2−^,Cp = C_5_H_5_^−^^43^.

### Magnetic properties

**Fig. 3:**
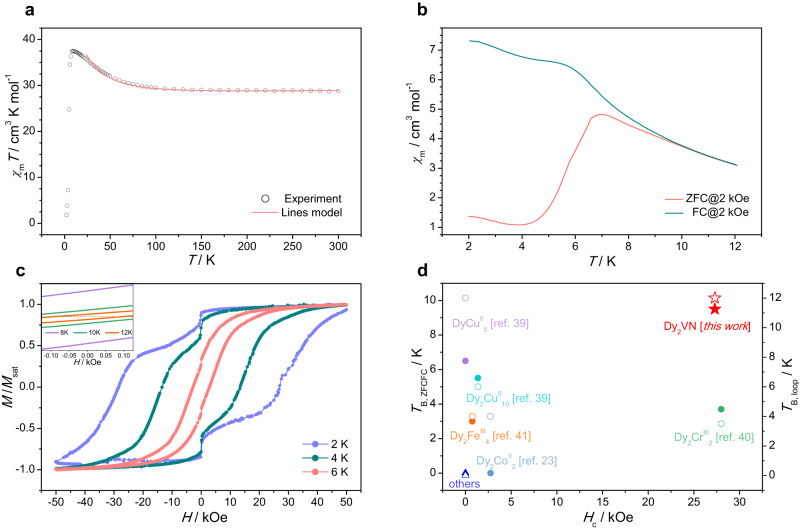
Magnetic properties of Dy_2_VN@*I*_*h*_(7)-C_80_. **a** Temperature dependence of *χ*_m_*T* products under 1 kOe dc field and the fitted temperature-dependent susceptibilities using Lines model. The experimental values were first scaled by a factor of 1.09 to make them consistent with the theoretical values at room temperature before fitting. **b** Zero-field-cooled (ZFC) magnetization and field-cooled (FC) magnetization under 2 kOe dc field at sweep rate of 3 K min^-1^. **c** Magnetic hysteresis at different temperatures with the sweep rate of 200 Oe s^-1^. The inset shows that the opening hysteresis can be observed at up to 12 K. **d** Comparison of the blocking temperature (*T*_B, ZFCFC_ and *T*_B, loop_) and coercive field (*H*_c_) between Dy_2_VN@*I*_*h*_(7)-C_80_ and typical 3d-4f SMMs^23,39–41^. The solid symbol represents *T*_B, ZFCFC_ while the hollow symbol corresponds to *T*_B, loop_. The pentacle represents Dy_2_VN@*I*_*h*_(7)-C_80_ in this work. The triangle stands for the SMMs in Table S6 for which no related values are given. The circles stand for the other SMMs shown in Table S6.

The magnetic properties of Dy_2_VN@*I*_*h*_(7)-C_80_ were measured on a Quantum Design MPMS3 SQUID magnetometer (Fig. 3 and Fig. S3-S6). The temperature-dependent susceptibility data were collected under 1 kOe direct current (dc) field on the powder sample in the temperature range of 2–300 K. On cooling, the *χ*_m_*T* values slightly go up from 26.4 cm^3^ K mol^−1^ at 300 K to 27.02 cm^3^ K mol^−1^ at 100 K and increase sharply with further cooling, reaching a maximum of 34.4 cm^3^ K mol^−1^ at around 10 K (Fig. 3a). The significant increase of *χ*_m_*T* values with temperature decreasing implies ferromagnetic interaction within the cluster. Below 10 K, the *χ*_m_*T* values drop rapidly due to the depopulation of the Stark levels and magnetic anisotropies as the temperature decreases. The *χ*_m_*T* value (26.4 cm^3^ K mol^−1^) at 300 K is slightly smaller than the theoretical value of 29.34 cm^3^ K mol^−1^ for two uncoupled Dy (III) ions (the ground state ^6^H_15/2_ and *g*_J_ = 4/3) and one V (III) ion (*S* = 1 and *g* = 2).

The zero-field-cooled (ZFC) magnetization and field-cooled (FC) magnetization (ZFC-FC) data were collected in the heating mode under 2 kOe dc field. The temperature of bifurcation in the ZFC-FC curve (*T*_B, ZFCFC_) of Dy_2_VN@*I*_*h*_(7)-C_80_ is determined to be 9.5 K **(**Fig. 3b), which offers the highest *T*_B_ in all reported 3d-4f SMMs (Fig. 3d and Table S6). In accordance with its *T*_B, ZFCFC_, Dy_2_VN@*I*_*h*_(7)-C_80_ exhibits open magnetic hysteresis up to 12 K (*T*_B, loop_), as described in Fig. 3c. The hysteresis is quite broad with a coercive field of 2.73 T at 2 K (Fig. 3d and Fig. S5). To the best of our knowledge, this is among the largest coercive fields in all reported 3d-4f SMMs^22–24,39–43^ (Fig. 3d and Table S6).

Spin dynamics of Dy_2_VN@*I*_*h*_(7)-C_80_ were also characterized by time-dependent dc measurements. Zero-field magnetization relaxation times *τ* below 15 K were determined by free-order exponential fitting of magnetization decay curves recorded after being magnetized by 1 kOe dc field (Fig. S6 and Table S7). The fitting of relaxation times *τ vs. T*^−1^ could be accomplished by combining Orbach and QTM processes using Eq. (1):

1 $${\tau }^{-1}={\tau }_{0}^{-1}\exp \left(-{U}_{1}/T\right)+{\tau }_{{{{{{\rm{QTM}}}}}}}^{-1}$$

**Fig. 4:**
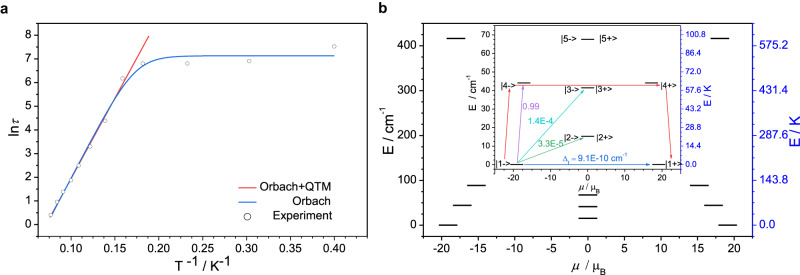
Exchange energy barriers of Dy_2_VN@*I*_*h*_(7)-C_80_. **a** Plots of the logarithm of the relaxation times (ln*τ*) vs. reciprocal temperature (*T*^-1^). The circles represent the relaxation times *τ* extracted from magnetization decay curves. The blue solid line indicates the fitting with the combination of Orbach and QTM processes, while the red line represents the result of only using Orbach process above 6 K (see in Supporting Information). **b** Energy levels obtained from Lines model. The black lines represent the pseudo-doublets as a function of their magnetic moments along the magnetic axis. Inset: low-lying energy levels of the exchange part. The number at each arrow stands for the mean absolute value of the corresponding matrix element of the transition magnetic moment. The red arrow corresponds to the deduced relaxation pathway (*U*_Lines_ = 63.4 K).

The best fit gives the Orbach barrier of *U*_1_ = 70.7 K (Fig. 4a), which is actually the exchange barrier. And the fitted QTM relaxation time is *τ*_QTM_ = 1249.8 s (Fig. 4a), indicating that QTM is effectively inhibited in Dy_2_VN@*I*_*h*_(7)-C_80_. *T*_B_ can also be obtained directly from the relaxation times. Sessoli et al. have suggested a more universal SMM characteristic, *T*_B, 100_, the temperature at which the relaxation time is 100 s^44^. However, for most of the reported SMMs, the relaxation times were usually much shorter than 100 s within the detectable temperature range. To the best of our knowledge, there is only one 3d-4f SMM [Dy_2_Cu^II^_10_(quinha)_10_(sal)_2_(OH)(py)_9_]^3+^ whose *T*_B, 100_ is *ca*. 2 K^39^. For Dy_2_VN@*I*_*h*_(7)-C_80_, *T*_B, 100_ deduced from the temperature dependence of *τ* reaches to *ca*. 7.2 K.

### Theoretical Analysis

The performance of polynuclear SMMs is affected not only by the magnetic anisotropy of single ion, but also the interactions between paramagnetic centers. Therefore, the exploration of magnetic interactions in polynuclear systems utilizing theoretical calculation is helpful to reveal the origin of magnetic relaxation. The studied molecule Dy_2_VN@*I*_*h*_(7)-C_80_ is a three-center spin system and the magnetic states can be described with the total spin Hamiltonian (Eq. 2):

2 $${\hat{H}}_{{tot}}=	 {\hat{H}}_{{{{{{\rm{CF}}}}}}({{{{{\rm{Dy}}}}}}1)}+{\hat{H}}_{{{{{{\rm{CF}}}}}}\left({{{{{\rm{Dy}}}}}}2\right)}+{\hat{H}}_{{{{{{\rm{ZFS}}}}}}({{{{{\rm{V}}}}}})}-{J}_{{{{{{\rm{Dy}}}}}},{{{{{\rm{Dy}}}}}}}{\hat{\,J}}_{{{{{{\rm{Dy}}}}}}1}\cdot {\hat{J}}_{{{{{{\rm{Dy}}}}}}2} \\ 	- {J}_{{{{{{\rm{Dy}}}}}},{{{{{\rm{V}}}}}}}\left({\hat{J}}_{{{{{{\rm{Dy}}}}}}1}\cdot {\hat{S}}_{{{{{{\rm{V}}}}}}}+{\hat{J}}_{{{{{{\rm{Dy}}}}}}2}\cdot {\hat{S}}_{{{{{{\rm{V}}}}}}}\right)+\sum {\hat{H}}_{{{{{{\rm{Zeeman}}}}}}}$$

where the first two terms describe the crystal-field (CF) effect of the Dy centers and the third stands for the zero-field splitting (ZFS) of the V center. The fourth and fifth terms describe the interactions between the magnetic centers while the last term represents the Zeeman interaction. The interactions between different Dy centers and V center are considered to be identical as the two Dy centers are structurally identical.

Before proceeding to the discussion of the interactions within the cluster and understanding the energy-level spectrum simulated with the Hamiltonian (Eq. 2), single-ion CF parameters for Dy center and ZFS parameters for V center need to be treated properly. Ab initio calculations were performed at the CASSCF/SO-RASSI level of theory^45^. The computational results show that both Dy centers have strong uniaxial magnetic anisotropy and the ground state easy-axis is aligned along the metal-nitrogen bond, as shown in Fig. S8. The calculated ground state *g* tensors of Dy centers are very close to the values of the Ising limit states and the overall crystal-field splitting is up to 1500 cm^−1^, with the first excited state of higher than 400 cm^−1^ (Table S10). In contrast, the calculated *E/D* for V center is 0.31, thus it could be treated as isotropic with an average *g*_iso_ of 1.9. The calculated high magnetic excited states of both single Dy center and V center indicate that the much lower *U*_1_ may stem from the magnetic coupling of spin centers within the fullerene cage.

With the corresponding magnetic properties of the mononuclear fragments in hand, the magnetic interactions were explored to further elucidate the magnetic relaxation of Dy_2_VN@*I*_*h*_(7)-C_80_. The temperature-dependent susceptibilities of Dy_2_VN@*I*_*h*_(7)-C_80_ were fitted based on Eq. (2) and only the data above 20 K were considered during the fitting (See in Methods, Fig. 3a). Given the fitting result from Table 2, the interaction between Dy centers is characterized as antiferromagnetic while the interaction between Dy and V is ferromagnetic. The exchange-coupled levels and *g*-tensor values of Dy_2_VN@*I*_*h*_(7)-C_80_ were also calculated and summarized in Table S11. These levels are grouped into pseudo-doublets split by the tunneling gap (*∆*_t_) because of the even number of unpaired electrons in Dy_2_VN@*I*_*h*_(7)-C_80_. The calculated ground exchange state features an extremely small *∆*_t_, indicating that QTM is suppressed and large *H*_c_ could be observed. On the contrast, the third excited pseudo-doublet possesses a relatively large *∆*_t_, thus the relaxation pathway could be deduced as indicated with red arrows in Fig. 4b combining with the transition probabilities. The inferred exchange barrier gives *U*_Lines_ = 63.4 K and is well consistent with the energy barrier fitted from the data of demagnetization (*U*_1_ = 70.7 K). Although the splitting of the energy states is much lower than the crystal-field splitting of an individual Dy(III), the strong coupling between Dy(III) and V(III) can effectively suppress QTM, leading to large coercivity and high *T*_B_.

**Table 2:** The magnetic interactions obtained using Lines model

	Dy-Dy	Dy-V
*J*_dip_ / cm^-1^	6.25	0.80
*J*_exch_ / cm^-1^	-12.5	52.50
*J*_total_ / cm^-1^	-6.25	53.30

## Discussion

Enhancing magnetic coupling of paramagnetic centers is an effective strategy for realizing excellent SMM behaviors. Strong interactions between the metal centers in Dy_2_VN@*I*_*h*_(7)-C_80_ bring about negligible transverse components of *g*-tensor (*g*_x_ = *g*_y_ = 0) and large magnetic moment of the ground state, which suppress QTM and lead to hard magnetism. In 2014, Greber et al.^46^ reported the magnetic properties of Dy_n_Sc_3-n_N@*I*_*h*_-C_80_ (n = 1, 2, 3) containing different numbers of paramagnetic Dy(III). The different hysteresis behaviors of Dy_n_Sc_3-n_N@*I*_*h*_-C_80_ (n = 1, 2, 3) revealed that the number of magnetic metal ions and their interactions have a significant influence on the properties of EMFs. In zero field, the magnetization of DySc_2_N@*I*_*h*_-C_80_ decays rapidly via quantum tunneling. For Dy_2_ScN@*I*_*h*_-C_80_, the demagnetization needs to overcome the exchange barrier or simultaneously reverse the magnetic moments of the two Dy(III) ions, which suppresses QTM and cause remanence. The much narrower hysteresis of Dy_2_ScN@*I*_*h*_-C_80_ in comparation to Dy_2_VN@*I*_*h*_(7)-C_80_ also demonstrates that the strong coupling (53.3 cm^−1^) between Dy(III) and paramagnetic V(III) is fundamental for magnetic relaxation, while in Dy_2_ScN@*I*_*h*_-C_80_ there is only a much weaker Dy-Dy interaction. The introduction of paramagnetic V(III) instead of diamagnetic Sc(III) could result in multilevel exchange states and suppress QTM, leading to larger *H*_c_ (0.5 T at 2 K with sweep rate at 80 Oe s^−1^ for Dy_2_ScN@*I*_*h*_-C_80_). The noncolinear ferromagnetic coupling among both paramagnetic centers in Dy_3_N@*I*_*h*_-C_80_ leads to a frustrated ground state, where the tunneling between six-fold degenerate ground states may facilitate demagnetization and cause much weaker remanence than in Dy_2_ScN@*I*_*h*_-C_80_. In contrast, replacing one Dy(III) with a V(III), the six-fold degeneration in Dy_3_N@*I*_*h*_-C_80_ was broken. Combining with the strong interaction between 3d-4f metals, the broken degeneration could effectively inhibit QTM, making Dy_2_VN@*I*_*h*_(7)-C_80_ one of the most remarkable SMMs.

The presence of metal bonds can effectively enhance magnetic coupling, and the three-spin [Ln^3+^-e-Ln^3+^] systems^28–30^ with strong coupling have shown promise as SMMs. Nevertheless, the larger magnetic moment of V(III) resulted in larger remanence of Dy_2_VN@*I*_*h*_(7)-C_80_ than Dy_2_@C_80_(CH_2_Ph) (1.15 T at 2 K with sweep rate at 29 Oe s^−1^) and Dy_2_@C_79_N (1.5 T at 2 K with sweep rate at 200 Oe s^−1^). In that way, paramagnetic 3d metals are of vital importance to 3d-4f SMM behavior and a diverse array of 3d-4f SMMs have been reported (Table S6). Generally speaking, the enhanced magnetic coupling between paramagnetic lanthanides and 3d ions can be an effective means to improve the performance of SMMs, while the unique confinement effect of fullerenes may compress the distance between spin centers and enhance coupling.

In summary, a metal cluster Dy_2_VN has been confined inside a C_80_ fullerene cage, forming a metallofullerene containing both paramagnetic 4f and 3d metal ions. The molecular structure and internal metal interactions of Dy_2_VN@*I*_*h*_(7)-C_80_ were systematically studied by X-ray crystallography. The unique confinement effect of the cage leads to strong internal Dy-Dy and Dy-V interactions, as reflected by the ultrashort Dy-Dy and Dy-V distances. As a result of the strong magnetic coupling inside the cage, Dy_2_VN@*I*_*h*_(7)-C_80_ shows a record-high blocking temperature among all reported 3d-4f SMMs and huge coercive field. Our work opens a new class of 3d-4f SMMs based on EMFs, with superior magnetic properties endowed by the unique confinement effect of the cage. The construction of this new class of 3d-4f SMMs can be achieved through a modular approach by concurrent metal encapsulation inside the fullerene cage. Considering the diversity of the cage geometry, cluster structure and exohedral chemical functionalization on the cage, the range of possibilities for the magnetic properties of such SMMs is extensive.

## Methods

### Synthesis

Dy_2_O_3_ and vanadium carbide were used as metal sources to synthesize the mixed nitride cluster fullerenes with embedded Dy and V metals. Raw soot of EMFs was obtained by an arc-discharge method. The soot was extracted with CS_2_. After the removal of CS_2_, the residue was re-dissolved in toluene and the solution was subjected to multi-stage high-performance liquid chromatographic (HPLC) separation. Details of the separation process are described in the Supporting Information (Fig. S1).

### X-ray crystallography

Single crystals of Dy_2_VN@*I*_*h*_(7)-C_80_ were obtained by layering a benzene solution of Ni (OEP) (OEP is the dianion of octaethylporphyrin) atop of a saturated CS_2_ solution of Dy_2_VN@*I*_*h*_(7)-C_80_ in a glass tube. After two weeks, the two solutions diffused together and black crystals formed. X-ray data were collected at 100 K using a radiation wavelength of 0.67012 Å at beamline BL17B of the Shanghai Synchrotron Radiation Facility. A multi-scan method was used for absorption corrections. The structures were solved with direct methods and were refined with SHELXL-2018. CCDC 2193585 contains the supplementary crystallographic data of this paper. Details of the structural refinement can be found in the Supporting Information.

### Magnetometry

Magnetic properties were determined using a Quantum Design MPMS3 magnetometer. DC mode was adopted for the measurements of susceptibility and magnetization, while VSM mode was selected for hysteresis, zero-field-cooled (ZFC) magnetization and field-cooled (FC) magnetization (ZFC-FC) and magnetization decay measurements. The sample (0.340 mg) was prepared by drop-casting from carbon disulfide solution onto a slice of Al foil (5.413 mg) which is paramagnetic to minimize the background of sample holder. Then, fast evaporation of carbon disulfide afforded black powder. After that, the Al foil was folded into a small cube and stuck on the inner wall of a plastic straw with a tiny amount of N grease (less than 1 mg). The background of Al foil and Pascal correction were considered when the point-by-point correction was carried on the data.

### Theoretical calculations

Ab initio calculations of the Dy_2_VN@*I*_*h*_(7)-C_80_ model systems have been performed at the CASSCF/SO-RASSI level of theory employing the quantum chemistry package MOLCAS 8.1^47^. Dynamic correlation energy was considered using the CASPT2 program for the calculation of V(III). The single-ion magnetic properties and CF/ZFS parameters were calculated with SINGLE_ANISO program^45,48,49^ based on the ab initio results. The calculation models were built on the basis of crystal structure and without further optimization. For the calculation of electronic structure of a single Dy center, the other Dy(III) was replaced by a diamagnetic Lu(III) while V(III) was replaced with a diamagnetic Sc(III) in the model molecule. Similarly, both Dy centers were replaced with Lu(III) in the magnetic calculation of V(III). The magnetic states of Dy_2_VN@*I*_*h*_(7)-C_80_ can be described with the total spin Hamiltonian (Eq. 2), where ${J}_{{{{{{\rm{Dy}}}}}},{{{{{\rm{Dy}}}}}}}$ represents total interactions (including dipolar and exchange interaction) between Dy centers and ${\hat{J}}_{{Dy}}$ is the localized dysprosium magnetic moment. Similarly, ${J}_{{{{{{\rm{Dy}}}}}},{{{{{\rm{V}}}}}}}$ represents total interactions between Dy and V, and ${\hat{S}}_{V}$ is the spin of V(III). With the corresponding magnetic properties of the mononuclear fragments in hand, the magnetic interactions were then explored. The anisotropic exchange interactions between the magnetic centers were considered within the Lines model^50^ using the Hamiltonian (Eq. 3):

3 $${\hat{H}}_{{{{{{\rm{exchange}}}}}}}=-{J}_{{{{{{\rm{exch}}}}}},{{{{{\rm{DyDy}}}}}}}{\widetilde{S}}_{{{{{{\rm{Dy}}}}}}1,{{{{{\rm{z}}}}}}}\cdot {\widetilde{S}}_{{{{{{\rm{Dy}}}}}}2,{{{{{\rm{z}}}}}}}-{J}_{{{{{{\rm{exch}}}}}},{{{{{\rm{DyV}}}}}}}\left({\widetilde{S}}_{{{{{{\rm{Dy}}}}}}1,{{{{{\rm{z}}}}}}}\cdot {\hat{S}}_{{{{{{\rm{V}}}}}}}+{\widetilde{S}}_{{{{{{\rm{Dy}}}}}}2,{{{{{\rm{z}}}}}}}\cdot {\hat{S}}_{{{{{{\rm{V}}}}}}}\right)$$

while the dipole−dipole magnetic coupling is treated exactly (see details in Supporting Information). The first term describes the exchange interaction between the two Dy centers and the second one corresponds to the exchange interaction between Dy and V center. The subscript of the pseudospin/spin operators denotes the local anisotropy axis on the corresponding metal center. The spin of V(III) was regarded as *S* = 1, while Dy(III) was treated with the pseudospin $\widetilde{S}$ = 1/2. The temperature-dependent susceptibilities of Dy_2_VN@*I*_*h*_(7)-C_80_ were fitted employing the POLY_ANISO program^48,49^.

### Supplementary information

Supplementary Information

Peer Review File

## Data Availability

All data supporting the findings of this study are available with the paper and its supplementary information files. The crystallographic data for Dy_2_VN@*I*_*h*_(7)-C_80_ in this study have been deposited at the Cambridge Crystallographic Data Centre under CCDC number 2193585 [10.5517/ccdc.csd.cc2cmltf]. The data are also available from the corresponding authors upon reasonable request.
