# Supplementary material for: A hard molecular nanomagnet from confined paramagnetic 3d-4f spins inside a fullerene cage
Source: Nat Commun. 2023 Dec 19;14:8443. doi: 10.1038/s41467-023-44194-y (PMC10730828; doi:10.1038/s41467-023-44194-y)
Supplement: Supplementary file 1 — Supplementary Information [file 41467_2023_44194_MOESM1_ESM.pdf]

Content

S1. General Instruments. S2

S2. HPLC Separation of $Dy_2VN@I_h(7)$-C_{80}. S2

S3. Crystal Structure of $Dy_2VN@I_h(7)$-C_{80}. S3

S4. Electronic Structure of $Dy_2VN@I_h(7)$-C_{80}. S5

S5. Magnetic Properties of $Dy_2VN@I_h(7)$-C_{80}. S6

S6. Theoretical Analysis S11

S7. Coordinates based on DFT optimization S17

S8. Reference S33

S1. General Instruments.

High-performance liquid chromatography (HPLC) was conducted on an LC-908 instrument (Japan Analytical Industry Co., Ltd.) with toluene as mobile phase. Matrix-assisted laser desorption ionization time-of-flight (MALDI-TOF) mass spectrometry was measured on a BIFLEX III spectrometer (Bruker Daltonics Inc., Germany). UV-Vis-NIR spectra were recorded from a PE Lambda 750S spectrophotometer with sample dissolved in toluene. Cyclic voltammograms (CV) was measured in *o*-dichlorobenzene with 0.05 M $TBAPF_6$ as supporting electrolyte at a Pt working electrode with a CHI660E workstation. Magnetic properties were determined using Quantum Design MPMS3 magnetometer.

S2. HPLC Separation of $Dy_2VN@I_h(7)$-C_{80}.

The entire separation process consists of four steps. The first stage was performed using a Buckyprep column with toluene as mobile phase (Fig. S1a). Fr-6 (retention time: 61-68 min) was collected in the first stage and re-injected into the recycling second stage using a Buckyprep-M column and toluene as the eluent (Fig. S1b). Fr6-4 (retention time: 134-138 min) was collected in the second stage and re-injected into the third recycling stage using a Buckyprep column and toluene as the eluent. Fr6-4-2 was collected as the final product $Dy_2VN@I_h(7)$-C_{80} (Fig. S1c).

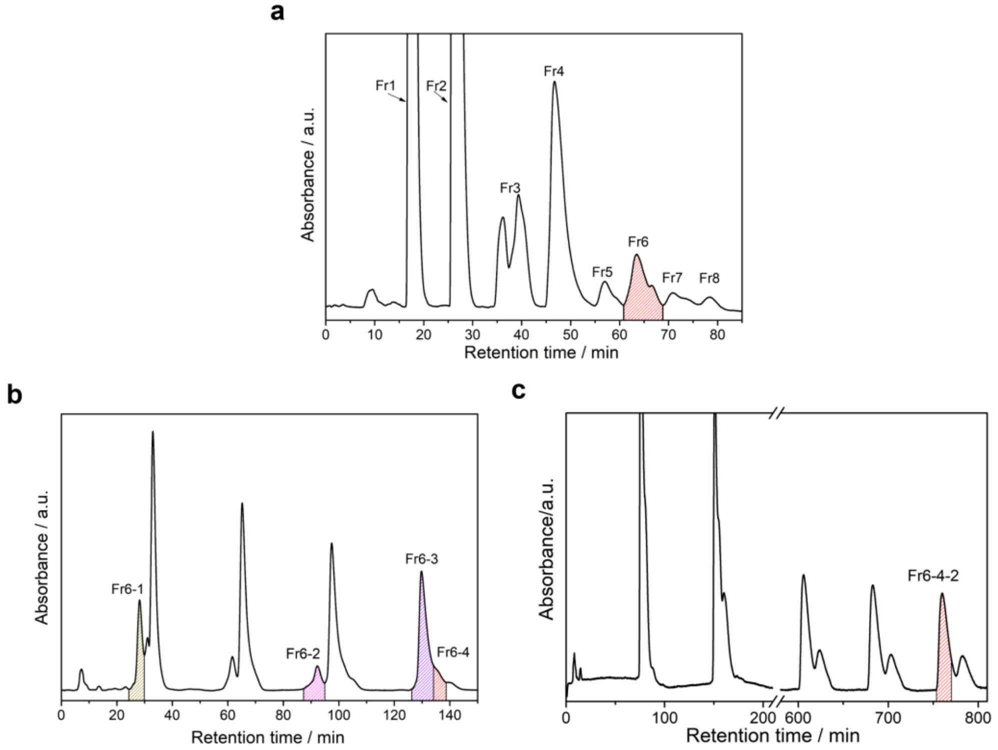

Supplementary Fig. S1. HPLC separation profiles of $Dy_2VN@I_h(7)$-C_{80}. **a**, The first stage HPLC chromatogram of the extract. HPLC conditions: Buckyprep column, Φ 20 mm × 250 mm, eluent = toluene, flow rate = 10 mL min^{-1}, detection wavelength = 330 nm. **b**, The recycling HPLC chromatogram of Fr6. HPLC conditions: Buckyprep-M column, Φ 20 mm × 250 mm, eluent = toluene, flow rate = 10 mL min^{-1}, detection wavelength = 330 nm; **c**, The recycling HPLC chromatogram of Fr6-4. HPLC conditions: Buckyprep column, Φ 20 mm × 250 mm, eluent = toluene, flow rate = 10 mL min^{-1}, detection wavelength = 330 nm.

S3. Crystal Structure of $Dy_2VN@I_h(7)$-C_{80}.

Supplementary Table S1. Crystal data of $Dy_2VN@I_h(7)$-C_{80}.

Compound	$Dy_2VN@I_h(7)$-$C_{80}\cdot Ni^{II}(OEP)\cdot C_6H_6$
Temperature, K	100(2)
Wavelength, Å	0.67012
Empirical formula	C250 H106 Dy4 N10 Ni2 V2
Formula weight	4118.74
Z	2
Crystal system	Monoclinic
Space group	*C2/m*
a, Å	25.1985(11)
b, Å	15.1525(7)
c, Å	19.7180(9)
α, deg	90
β, deg	95.339(2)
γ, deg	90
Volume, $Å^3$	7496.1(6)
Density, mg cm^{-3}	1.825
Absorption coefficient, mm^{-1}	2.095
F(000)	4084
Crystal size, mm^3	0.200 × 0.200 × 0.150
Data/restraints/parameters	7133/715/696
Reflections collected	47589
Independent reflections	7133 [*R*(int) = 0.1107]
Goodness-of-fit on F^2	1.066
Final R indices [*I*>2sigma(*I*)]	R_1 = 0.1348, wR_2 = 0.3270
R indices (all data)	R_1 = 0.1861, wR_2 = 0.3972

The shortest distance between the Ni atom in the porphyrin unit to the carbon cage is 2.954 Å, indicating strong π-π interactions between $Dy_2VN@I_h(7)$-C_{80} and Ni (OEP). Inside the cage, both Dy and V atoms have multiple disordered sites. Fig. S2 shows the specific positions of disordered metal atoms inside the carbon cage. In the structure of $Dy_2VN@I_h(7)$-$C_{80}\cdot$Ni (OEP)$\cdot C_6H_6$, six disordered Dy ions were found in the crystal structure, which were denoted as Dy1, Dy2, Dy3, Dy4, Dy5, and Dy6, respectively. After the crystallographic symmetric operation, six disordered Dy sites named Dy1A, Dy2A, Dy3A, Dy4A, Dy5A, and Dy6A are respectively obtained. Compared with Dy ions, V has a lower degree of disorder. Two disordered V ions were found in the crystal structure analysis. V1 are located on the crystallographic mirror plane which has the highest occupancy of

0.44076. V2A is obtained by crystallographic symmetric operation of V2.

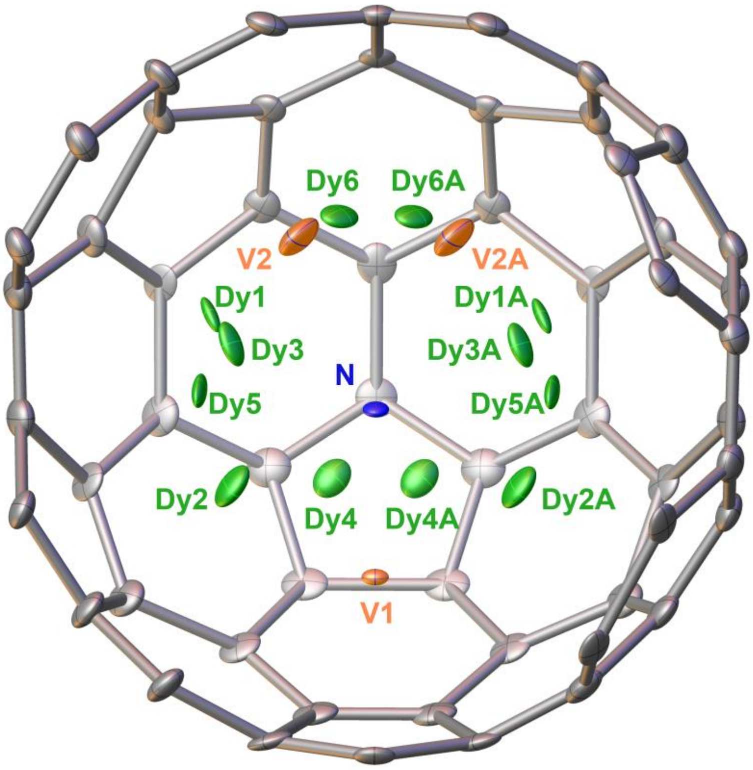

Supplementary Fig. S2. Disordered sites of internal Dy and V atoms in $Dy_2VN@I_h(7)$-C_{80}. Selected cage carbon atoms have been omitted for clarity.

Supplementary Table S2. Occupancy values of the inner Dy_2VN cluster.

Disordered site	Occupancy	Disordered site	Occupancy
Dy1	0.42758	V1	0.44076
Dy1A	0.42758	V2	0.27962
Dy2	0.15661	V2A	0.27962
Dy2A	0.15661		
Dy3	0.11839		
Dy3A	0.11839		
Dy4	0.05432		
Dy4A	0.05432		
Dy5	0.15023		
Dy5A	0.15023		
Dy6	0.09287		
Dy6A	0.09287		

S4. Electronic Structure of $Dy_2VN@I_h(7)\text{-}C_{80}$.

Supplementary Table S3. The characteristic absorption peaks, absorption onset, and optical bandgap of $Dy_2VN@I_h(7)\text{-}C_{80}$, $Dy_xSc_{3\text{-}x}N@I_h(7)\text{-}C_{80}$ (x = 1-3), and $V_xSc_{3\text{-}x}N@I_h(7)\text{-}C_{80}$ (x = 1-2).

EMFs	Absorption peaks / nm	Absorption onset / nm	$\Delta E_{gap,\ optical}$ / eV[a]	Ref.
$Dy_2VN@I_h(7)\text{-}C_{80}$	465, 581, 663, 773	1424	0.87	*This work*
$Dy_2ScN@I_h(7)\text{-}C_{80}$	406, 565, 676, 705	850	1.46	[S1]
$Dy_3N@I_h(7)\text{-}C_{80}$	320, 401, 554, 626, 643, 670, 700	823	1.51	[S2]
$V_2ScN@I_h(7)\text{-}C_{80}$	327, 603	1290	0.96	[S3]
$VSc_2N@I_h(7)\text{-}C_{80}$	313, 358, 748	1700	0.73	[S3]
$Dy_2ScN@D_{5h}(6)\text{-}C_{80}$	416, 458, 636, 722	980	1.26	[S4]
$DySc_2N@D_{5h}(6)\text{-}C_{80}$	408, 449, 641, 730	960	1.29	[S4]
$Dy_3N@D_{5h}(6)\text{-}C_{80}$	463, 627, 714	929	1.33	[S2]
$V_2ScN@D_{5h}(6)\text{-}C_{80}$	419, 481	1660	0.75	[S5]
$VSc_2N@D_{5h}(6)\text{-}C_{80}$	413, 475	1900	0.65	[S5]

[a] optical bandgap: $\Delta E_{gap,\ optical} = 1240 / \lambda_{onset}$.

The electrochemical properties of $Dy_2VN@I_h(7)\text{-}C_{80}$ were studied by cyclic voltammetry with tetrabutylammonium hexafluorophosphate ($TBAPF_6$) as the supporting electrolyte. The characteristic redox potentials are listed in Table S4. $Dy_2VN@I_h(7)\text{-}C_{80}$ has one reversible oxidation process and four reduction processes, in which the first, third and fourth reduction processes are all reversible, and the second reduction process is irreversible. The first oxidation potential and the first reduction potential are 0.05 V and -0.81 V, respectively, the electrochemical bandgap is 0.86 eV, which is in great agreement with its optical bandgap. In comparison to $Sc_2VN@I_h(7)\text{-}C_{80}$, both the oxidation potential and reduction potential of $Dy_2VN@I_h(7)\text{-}C_{80}$ move to the negative direction, suggesting a stronger electron-donating properties of $Dy_2VN@I_h(7)\text{-}C_{80}$. This could be rationalized by considering the larger ionic radius of Dy than Sc and the presumably resulted stronger metal-cage interactions in $Dy_2VN@I_h(7)\text{-}C_{80}$ than $Sc_2VN@I_h(7)\text{-}C_{80}$.

Supplementary Table S4. Redox potential table of $Dy_2VN@I_h(7)\text{-}C_{80}$, and $Sc_2VN@I_h(7)\text{-}C_{80}$.

**EMF**	**$E_{1/2}$ (V vs. Fc/Fc$^+$)**							**ΔE_{gap} (V)**
	$^{ox}E_2$	$^{ox}E_1$	$^{red}E_1$	$^{red}E_2$	$^{red}E_3$	$^{red}E_4$	$^{red}E_5$	
$Dy_2VN@I_h(7)\text{-}C_{80}$		0.05	-0.81	-1.53*	-1.81	-2.35		0.86
$Sc_2VN@I_h(7)\text{-}C_{80}$		0.44*	-0.42	-0.66	-1.33	-1.71	-2.32*	0.96
$Sc_2VN@D_{5h}(6)\text{-}C_{80}$	0.93*	0.42*	-0.78*	-1.59*	-2.01*	-2.37*		1.20

*irreversible procedure

S5. Magnetic Properties of $Dy_2VN@I_h(7)-C_{80}$.

Sample Preparation. The sample (0.340 mg) for magnetic measurements was prepared by drop-casting carbon disulfide (CS_2) solution onto a slice of Al foil (5.413 mg) which is paramagnetic to minimize the background of sample holder. Then, fast evaporation of CS_2 afforded black powder. After that, the Al foil was folded into a small cube and stuck on the inner wall of a plastic straw with very small amount of N grease (less than 1 mg). The mass of the sample was determined by the variation of the Al foil before and after drop-casting. All the mass values were weighed using a Metallo Toledo Ultra-micro balance (1 μg).

Magnetic measurements. Magnetic properties were determined using Quantum Design MPMS3 magnetometer. DC mode was adopted for the measurements of susceptibility and magnetization, while VSM mode was selected for hysteresis, zero-field-cooled (ZFC)/field-cooled (FC) magnetization (ZFC-FC) and magnetization decay measurements. The background of Al foil and Pascal correction were considered when the point-by-point diamagnetic correction was carried on the data. Due to the quantity limitation, alternative current (ac) susceptibility measurement was not conducted.

Relaxation times extraction from decay measurements. Considering the long magnetic relaxation times of $Dy_2VN@I_h(7)-C_{80}$, the relaxation times were determined from the magnetization decay measurements. The sample was first magnetized under 1 kOe dc field for saturation. Then the field was swept to zero as fast as possible and then the decay data was collected. The relaxation times were obtained by fitting the data using equation S1[S6,S7], where M_{eq}, M_0, τ and b are fitting parameters. Figure S5 shows the selected magnetization decay curves and their fittings. Table S7 shows the fitted relaxation times τ under different temperatures.

$$\mathrm{M}(t) = M_{eq} + \left(M_0 - M_{eq}\right)exp\left[-\left(\frac{t}{\tau}\right)^b\right] \qquad \text{(S1)}$$

Determination of the effective energy barrier (U_{eff}). The fitting of the whole dataset of relaxation times τ *vs.* T^{-1} could be accomplished by combining Orbach and quantum tunneling of magnetization (QTM) processes using equation S2. The best fit gives the Orbach barrier of U_1 = 70.7 K and the QTM relaxation time of τ_{QTM} = 1249.8 s. The magnetic relaxation in high-temperature range is usually dominated by the Orbach process, thus we also only fit the relaxation times τ *vs.* T^{-1} above 6 K. And only Arrhenius fitting of the dataset of relaxation times τ *vs.* T^{-1} above 6 K using equation S3 gives the effective exchange barrier of 68.0 K, which is consistent with U_1.

$$\tau^{-1} = \tau_0^{-1}\exp(-U_1/T) + \tau_{\mathrm{QTM}}^{-1} \qquad \text{(S2)}$$

$$\tau^{-1} = \tau_0^{-1}exp(-\frac{U_{\mathrm{eff}}}{T}) \qquad \text{(S3)}$$

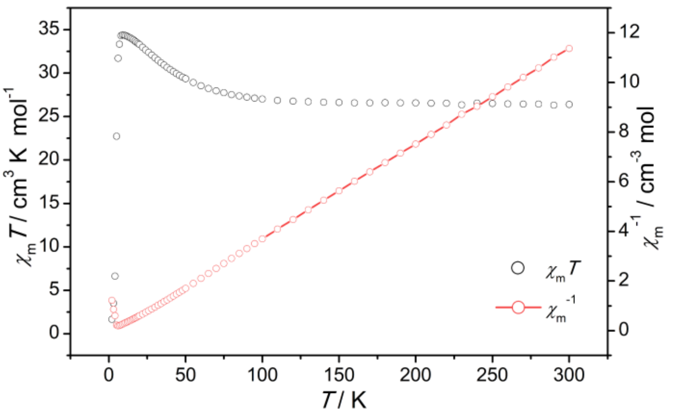

Supplementary Fig. S3. Temperature dependence of $\chi_m T$ and χ_m^{-1} for $Dy_2VN@I_h(7)$-C_{80} at 1 kOe dc field.

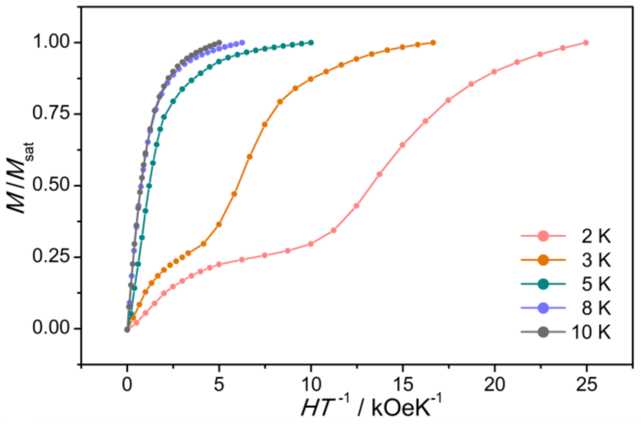

Supplementary Fig. S4. Plots of M vs. HT^{-1} at the temperatures of 2 K, 3 K, 5 K, 8 K and 10 K, respectively.

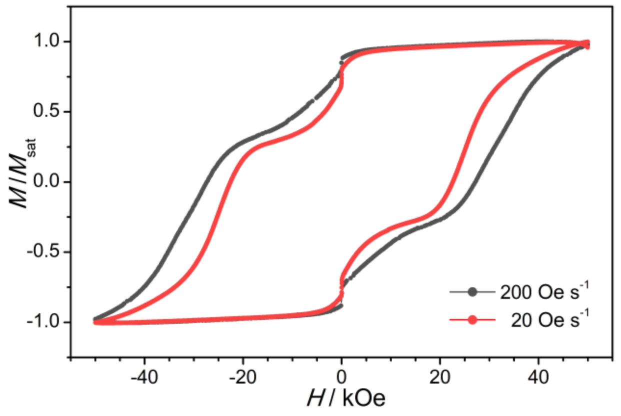

Supplementary Fig. S5. Magnetic hysteresis for $Dy_2VN@I_h(7)$-C_{80} at 2 K with different field sweep rates.

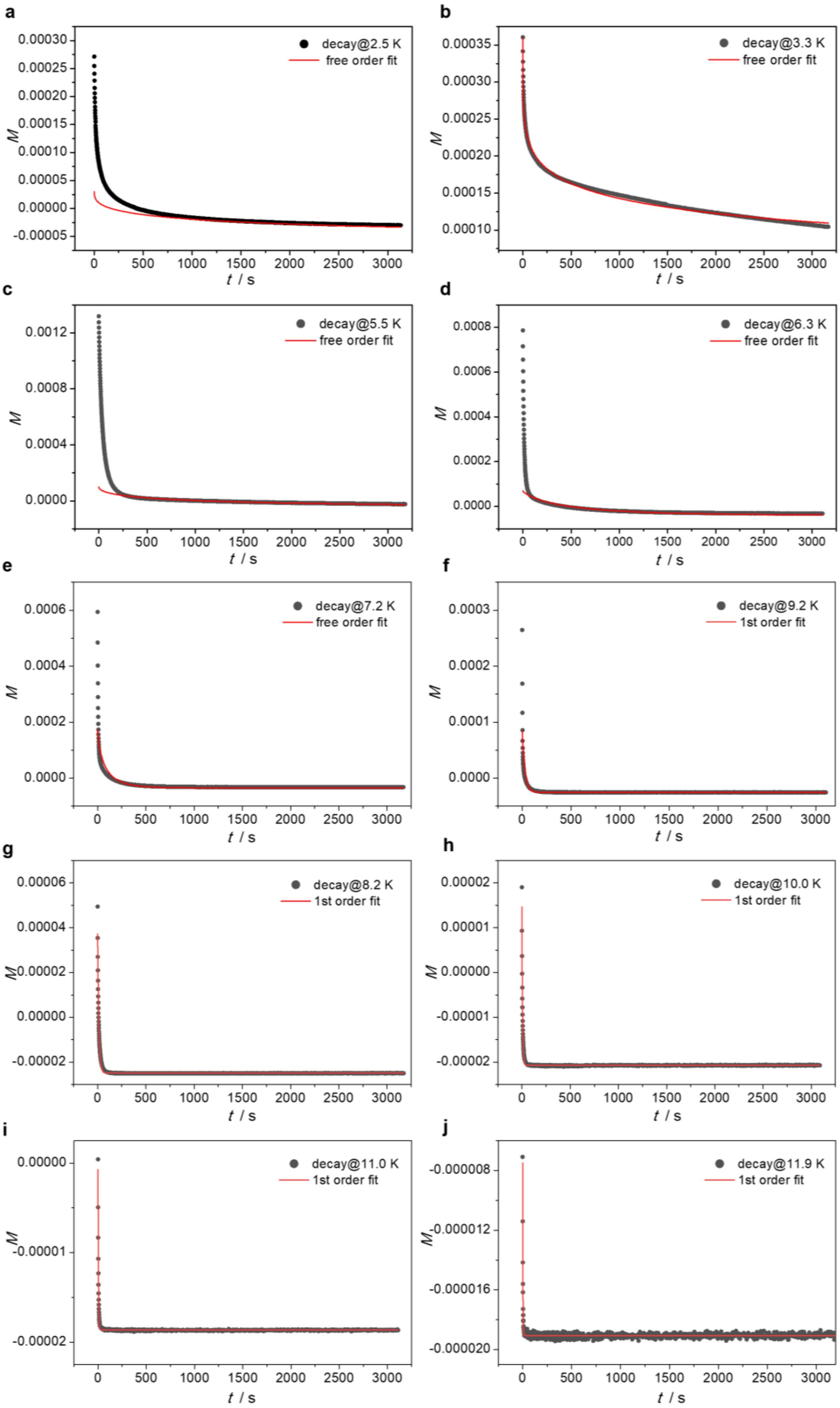
a
decay@2.5 K
free order fit
b
decay@3.3 K
free order fit
c
decay@5.5 K
free order fit
d
decay@6.3 K
free order fit
e
decay@7.2 K
free order fit
f
decay@9.2 K
1st order fit
g
decay@8.2 K
1st order fit
h
decay@10.0 K
1st order fit
i
decay@11.0 K
1st order fit
j
decay@11.9 K
1st order fit
M
t / s

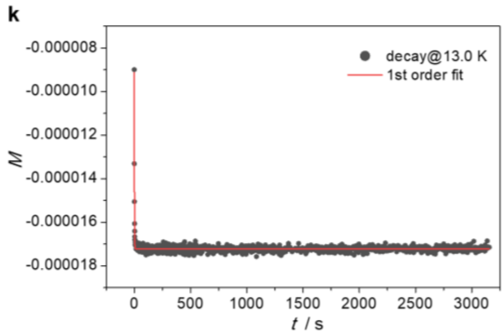

Supplementary Fig. S6. Determination of magnetization relaxation times from the magnetization decay curve at different temperatures for $Dy_2VN@I_h(7)\text{-}C_{80}$.

Supplementary Table S6. The blocking temperatures of different definition ($T_{B,\ ZFCFC}$ (K), and $T_{B,\ loop}$ (K)), coercive fields (H_c (kOe)) and magnetic interactions (J (cm^{-1})) for the selected 3d-4f SMMs.

	$T_{B,\ ZFCFC}$ / K	$T_{B,\ loop}$ / K	H_c / kOe	J / cm^{-1}	Ref.
$Dy_2V^{III}N$	9.5 (2 kOe,3 K min^{-1})[a]	12<br>(200 Oe s^{-1})	27.3 (2 K, 200 Oe s^{-1})<br>& 22.6 (2 K, 20 Oe s^{-1})	$J_{Dy\text{-}V}$ =53.30<br>$J_{Dy\text{-}Dy}$ = -6.25	*this work*
$DyCu^{II}_5$	6.5 (2 kOe,2 K min^{-1})[b]	12<br>(200 Oe s^{-1})	butterfly-shaped<br>(2 K, 200 Oe s^{-1})	$J_{Dy\text{-}Cu}$ =1.5<br>$J_{Cu\text{-}Cu}$ = -108.8	[S8]
$Dy_2Cu^{II}_{10}$	5.5 (2 kOe,2 K min^{-1})[b]	6<br>(200 Oe s^{-1})	1.37 (2 K, 200 Oe s^{-1})	$J_{Dy\text{-}Cu}$ =5.0 [c]<br>$J_{Cu\text{-}Cu}$ = -108.8 [c]<br>$J_{Dy\text{-}Dy}$ = 4.4 [c]	[S8]
$Dy_2Cr^{III}_2$	3.7<br>(1 kOe)[d]	3.5<br>(30 Oe s^{-1})	28 (1.8 K, 30 Oe s^{-1})	$J_{Dy1\text{-}Dy2}$ =-1.5<br>$J_{Cr1\text{-}Cr2}$ = 0.12<br>$J_{Dy1\text{-}Cr1}$ = -20.3<br>$J_{Dy1\text{-}Cr2}$ = -16.7	[S9]
$Dy_2Fe^{III}_4$	3<br>(1 kOe) [d]	4<br>(200 Oe s^{-1})	0.5 <H_c < 1<br>(2 K, 200 Oe s^{-1})	—	[S10]
$Dy_2Co^{II}_2$	—	4<br>(2350 Oe s^{-1})	2.5 <H_c < 5<br>(1.5 K, 2350 Oe s^{-1})	$J_{Dy\text{-}Co}$ =1.6<br>$J_{Co\text{-}Co}$ = 2.0	[S11]
$DyFe^{II}_2$	—	—	—	$J_{Dy\text{-}Fe1}$ =0.38<br>$J_{Dy\text{-}Fe2}$ = 0.06<br>$J_{Fe1\text{-}Fe2}$ = 0.09	[S12]
$Dy_2Mn^{II}_2$	—	—	—	$J_{Dy\text{-}Mn}$ =4.8<br>$J_{Mn\text{-}Mn}$ = 0.2<br>$J_{Dy\text{-}Dy}$ = 5.5	[S13]
$DyV^{IV}O$	—	—	—	$J_{Dy\text{-}V}$ = -0.254	[S14]

$Dy_2V^{III}N$=$Dy_2V^{III}N$@C_{80}; $DyCu^{II}_5$=[$DyCu^{II}_5$(quinha)$_5$(sal)$_2$(py)$_5$]-(CF_3SO_3)·py·4H_2O;[S8] $Dy_2Cu^{II}_{10}$=[$Dy_2Cu^{II}_{10}$(quinha)$_{10}$ (sal)$_2$(OH)(py)$_9$] (CF_3SO_3)$_3$·2py·2CH_3OH· 2H_2O; [S8] $Dy_2Cr^{III}_2$=$Cr^{III}_2Dy_2$(OMe)$_2$(O_2CPh)$_4$(mdea)$_2$(NO_3)$_2$; [S9] $Dy_2Fe^{III}_4$=[$Fe^{III}_4Dy_2$(μ_3-OH)$_2$ (mdea)$_6$(SCN)$_2$(NO_3)$_2$(H_2O)$_2$]·4H_2O·2MeCN; [S10] $Dy_2Co^{II}_2$=[$Co^{II}_2Dy_2$(L)$_4$(NO_3)$_2$(THF)$_2$] ·4THF, H_2L=(*E*)-2-(2-hydroxy-3-methoxybenzylideneamino)phenol; [S11] $DyFe^{II}_2$=[Fe^{II}_2Dy(L)$_2$(H_2O)]ClO_4·2H_2O, L=v2,2′,2″-(((nitrilotris(ethane-2,1-diyl))tris(azanediyl))tris(methylene))tris(4-chlorophenol)]; [S12] $Dy_2Mn^{II}_2$=$Dy_2Mn^{II}_2$(L)$_4$(NO_3)$_2$(DMF)$_2$, H_2L = (*E*)-2-ethoxy-6-(((2-hydroxyphenyl)imino)methyl)phenol; [S13] $DyV^{IV}O$=Dy(V^{IV}O)L(NO_3)$_3$(H_2O), H_2L = N, N′-bis(1-hydroxy-2-benzylidene-6-methoxy)-1,7-diamino-4-azaheptane. [S14]

a. The criterion of diverge temperature defined herein is 1% relative difference between FC and ZFC curves, $\frac{\chi_{FC}-\chi_{ZFC}}{\chi_{ZFC}} > 1\%$.

b. The criterion of diverge temperature defined herein is 5% relative difference between FC and ZFC curves. If the same criterion is used for Dy_2VN@I_h(7)-C_{80}, the $T_{B,\ ZFCFC}$ will be 9.1 K.

c. The $J_{Cu\text{-}Cu}$ was obtained from the fitting of [$DyCu_5$(quinha)$_5$(sal)$_2$(py)$_5$]-(CF_3SO_3)·py·4H_2O, while $J_{Dy\text{-}Cu}$ and $J_{Dy\text{-}Dy}$ were the effective Ising exchange interaction.

d. The temperature sweep rate was not indicated.

Supplementary Table S7. The extracted relaxation times (τ) and parameter b under different temperatures from magnetization decay.

T / K	T^{-1} / K^{-1}	τ / s	b
13.0	0.07692	1.5	1.0
11.9	0.08403	2.6	1.0
11.0	0.09091	4.0	1.0
10.0	0.10000	6.5	1.0
9.2	0.10870	12.1	1.0
8.2	0.12195	27.0	1.0
7.2	0.13889	80.0	0.73
6.3	0.15873	480	0.75
5.5	0.18182	900	0.52
3.3	0.30303	998	0.23
2.5	0.40000	1850	0.57

S6. Theoretical Analysis.

***Ab initio* calculations.** *Ab initio* calculations were performed at the CASSCF/SO-RASSI level of theory with the use of SINGLE_ANISO program[S15–S17] employing MOLCAS 8.1 program[S18] for both magnetic centers to obtain the crystal-field/zero-field parameters for Dy(III) and V(III). The calculation models were built on the crystal structure without further optimization. For the calculation of Dy(III), the other Dy(III) was replaced by diamagnetic Lu(III) and V(III) was replaced by diamagnetic Sc(III). The basis sets for all atoms are atomic natural orbitals from the MOLCAS ANO-RCC library[S19,S20]: ANO-RCC-VTZP for Dy(III)/V(III) ions; VTZ for close N and C; VDZ for distant C and diamagnetic Lu(III)/Sc(III). The calculations employed the second order Douglas-Kroll-Hess Hamiltonian, where scalar relativistic contractions were considered in the basis sets and the spin-orbit couplings were handled in the restricted active space state interaction (RASSI-SO) procedure. We have mixed the maximum number of spin-free states which was possible with our hardware (all from 21 sextets, 128 from 224 quadruplets, 130 from 490 doublets for the Dy(III) fragment). Active electrons in seven active spaces include all f electrons (CAS (9, 7)) of Dy (III) in the CASSCF calculation. For the calculation of V(III), two active electrons in five active spaces (CAS (5,2)) was adopted in the CASSCF calculation. Dynamic correlation energy was also be considered using the CASPT2[S21] program.

Density functional theory calculations. DFT calculations were performed at the B3LYP, PBE0, TPSSh, and ωB97X-D functionals with the basis function of 6-31G(d) for carbon atom and CEP-4G for metal atoms by using the quantum chemistry package Gaussian 16.[S22] The natural bond orbital calculations were performed with NBO 3.0 program[S23] also included in Gaussian 16.

The optimized results in Table S8 indicate the spin-ground state of $Dy_2VN@I_h(7)\text{-}C_{80}$ with $2S + 1 = 13$. The crystal structure of $Dy_2VN@I_h(7)\text{-}C_{80}$ is in line with the optimized one (Fig. S6) at the ωB97X-D/6-31G(d)~CEP-4G, which was considered to further understand the electronic structures. The Mulliken atomic spin population in Table S9 means that there are about 2, 5, and 5 unpaired electrons remain at V, Dy1, and Dy1A atoms, respectively, confirmed with the plot of spin density of $Dy_2VN@I_h(7)\text{-}C_{80}$ in Fig. S8. On the other hand, in comparison of ground-state electronic configuration of Dy ($4f^{10}6s^2$) and Sc ($3d^34s^2$) atoms, the NPA results indicate the three-electron

transfer of each metal atom leading to the formally six-electron transfer from inner cluster to outer fullerene cage, and there are clear back-donation in 3d and 5d for V and Dy atoms, respectively. The formally electronic structure can be characterized as $[(Dy^{3+})_2V^{3+}N^{3-}@I_h(7)\text{-}C_{80}^{6-}]$. Additionally, the metal-nonmetal interaction in inner cluster show clear covalent characters based on the Wiberg bond order calculations.

Supplementary Table S8. Relative energy (ΔE in kcal·mol^{-1}) of optimized $Dy_2VN@I_h(7)\text{-}C_{80}$ with different spin multiplicities ($2S + 1$) at different density functionals and same basis function 6-31G(d)~CEP-4G where S is the spin angular momentum.

**2S + 1**	ΔE/kcal·mol^{-1} **B3LYP**	ΔE/kcal·mol^{-1} **PBE0**	ΔE/kcal·mol^{-1} **TPSSh**	ΔE/kcal·mol^{-1} **ωB97X-D**
	6-31G(d)~CEP-4G			
1	144.5			
3	80.9			
5	42.4			
7	16.7			
9	0.0	1.6	0.3	3.8
11	7.4			
13	0.4	0.0	0.0	0.0
15	15.1			
17	56.0			
19	107.6			

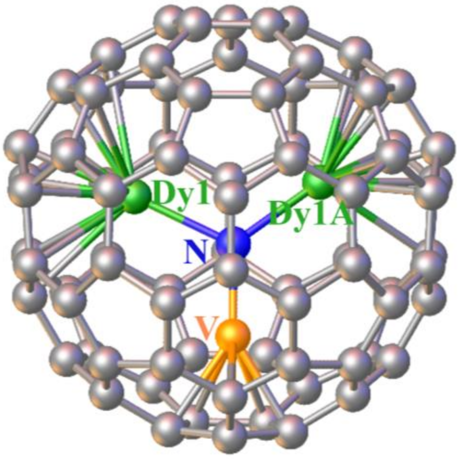

V-N: 1.790 Å (WBO: 1.78)
Dy1-N: 2.139 Å (WBO: 0.88)
Dy1A-N: 2.143 Å (WBO: 0.88)
V-Dy1: 3.491 Å (WBO: 0.20)
V-Dy1A: 3.331 Å (WBO: 0.24)
Dy1-Dy1A: 3.631 Å (WBO: 0.09)
∠V-N-Dy1A: 115.474°
∠V-N-Dy1: 125.105°
∠Dy1-N-Dy1A: 115.949°

Supplementary Fig. S7. Optimized geometry of $Dy_2VN@I_h(7)\text{-}C_{80}$ with $2S + 1 = 13$ spin-ground state at the ωB97X-D/6-31G(d)~CEP-4G, where the basis function of 6-31G(d) for carbon atom and CEP-4G for metal atoms, including the bond distance, bond angle, and Wiberg bond order (WBO) from mixed α and β density matrix.

Supplementary Table S9. Mulliken atomic spin population and natural population analysis (NPA) of Dy_2VN in optimized $Dy_2VN@I_h(7)\text{-}C_{80}$ with spin-ground state at the ωB97X-D/6-31G(d)~CEP-4G , where the basis function of 6-31G(d) for carbon atom and CEP-4G for metal atoms.

Atoms	Mulliken atomic spin population	NPA
Cage	-0.16	-
N	-0.12	$2s^{1.52}2p^{4.72}$
V	2.09	$3d^{3.57}4s^{0.14}4p^{0.37}4d^{0.20}$
Dy1	5.09	$4f^{9.02}5d^{0.94}6s^{0.09}6p^{0.28}$
Dy1A	5.10	$4f^{9.02}5d^{0.93}6s^{0.09}6p^{0.29}$

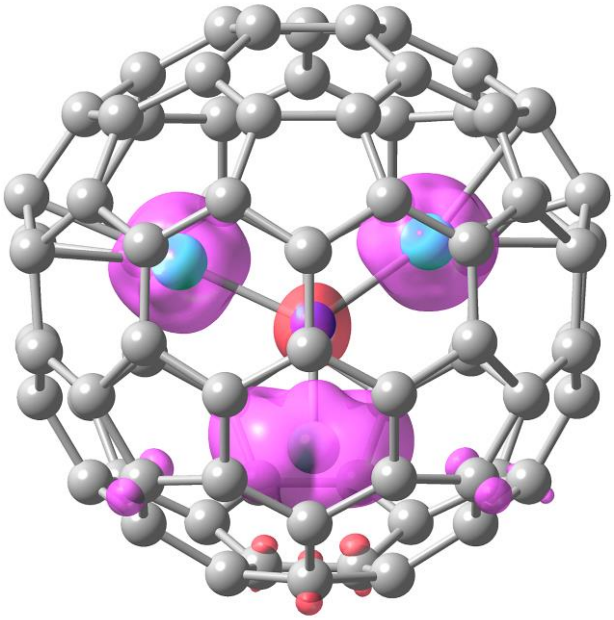

Supplementary Fig. S8. Plot of spin density for the optimized $Dy_2VN@I_h(7)\text{-}C_{80}$ with $2S + 1 = 13$ spin-ground state at the ωB97X-D/6-31G(d)~CEP-4G, where the basis function of 6-31G(d) for carbon atom and CEP-4G for metal atoms. The isovalue for the surface is 0.005 a.u.

Magnetic interaction fitting. The magnetic states of this three-center spin system can be described with the total spin Hamiltonian (Equation 2) as mentioned in the main text. The calculated crystal field parameters were employed to describe the crystal-field splitting of the single Dy(III) ion. Both the coupling within two Dy centers and the interaction between the Dy center and V center were included into the spin Hamiltonian. The two Dy centers are structurally identical as V–N locates on the crystallographic symmetric plane, thus the interactions between different Dy centers and V center can be considered to be identical.

According to the results from *ab initio*, the magnetic anisotropy of Dy centers are very close to the Ising limit, while the calculated E/D for V center is 0.31, thus the V center could be treated as isotropic with the average $g_{iso} = 1.9$. Therefore, the exchange interaction between the magnetic centers was considered within the Lines model using the Hamiltonian (Equation 3) in main text, while the dipole−dipole magnetic coupling is treated exactly with the Hamiltonian (Equation S4) as shown below where $\vec{r}$ is a unit vector and $|r|$ is the distance between the magnetic centers.

$$J_{\mathrm{dip}} = \frac{\mu_0 \mu_{\mathrm{B}}^2}{4\pi |r|^3} \cdot [\overline{g_1} \cdot \overline{g_2} - 3(\overline{g_1} \cdot \vec{r})(\vec{r} \cdot \overline{g_2})] \qquad \text{(S4)}$$

The fitting was conducted in the POLY_ANISO program[16,17] and only data above 20 K were considered during the fitting. The best fit gave $U_{1,\ \mathrm{Lines}}$ = 63.4 K and was well consistent with the energy barrier fitted from data of demagnetization (U_1 = 70.7 K).

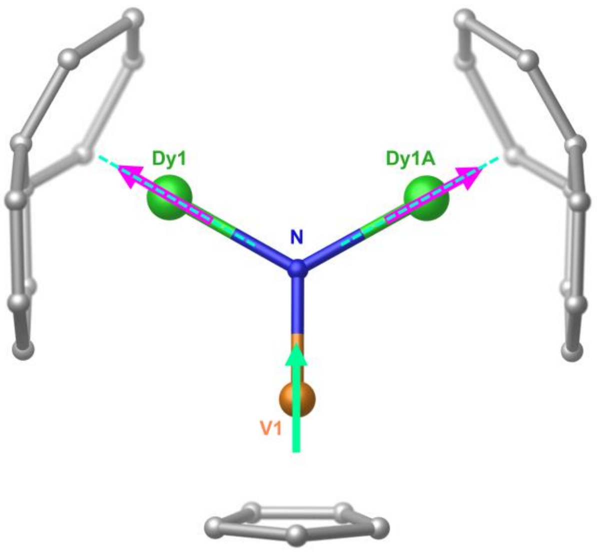

Supplementary Fig. S9 Orientation of easy axis (dashed lines) of the ground Kramers doublets on two Dy(III) centers and one possible representation of the orientation of the magnetic moments (arrows) on the paramagnetic centers for the ground state in $Dy_2VN@I_h(7)$-C_{80}. Dy: green; N: blue; V: orange.

Supplementary Table S10. Calculated energy levels and g (g_x, g_y, g_z) tensors of the lowest eight Kramers doublets (KDs) on individual Dy(III), and zero-field splitting parameters D, E and g (g_x, g_y, g_z) tensors of the lowest spin-orbit state on individual V(III) in $Dy_2VN@I_h(7)$-C_{80}.

KDs	Dy1			Dy1A			V1		
	E / cm^{-1}	E / K	g	E / cm^{-1}	E / K	g	D	E	g
1	0.0	0.0	0.000<br>0.000<br>19.852	0.0	0.0	0.000<br>0.000<br>19.844	1.8093 cm^{-1}	0.5593 cm^{-1}	1.856<br>1.908<br>1.961
2	414.1	595.5	0.004<br>0.004<br>17.041	414.0	595.4	0.004<br>0.004<br>17.028			
3	820.6	1180.0	0.069<br>0.076<br>14.261	820.5	1179.8	0.069<br>0.075<br>14.247			
4	1128.3	1622.5	0.149<br>0.270<br>11.471	1128.2	1622.3	0.146<br>0.267<br>11.458			

			0.985			0.980
5	1276.6	1835.8	1.849	1276.5	1835.6	1.838
			12.438			12.390
			3.452			3.452
6	1345.1	1934.2	5.085	1345.0	1934.0	5.055
			10.644			10.584
			1.359			1.360
7	1415.8	2036.0	2.884	1415.7	2035.8	2.871
			15.008			14.940
			0.161			0.161
8	1549.1	2227.7	0.441	1549.0	2227.5	0.437
			19.284			19.184

Supplementary Table S11. The obtained energy states based on Lines model and their corresponding *g*-tensor values.

	E / cm^{-1}	E / K	Δ_t / cm^{-1}	g_x	g_y	g_z
1	0.0	0.0	9.1E-10	0.000	0.000	38.23
2	15.3	22.0	2.0E-09	0.000	0.000	23.83
3	41.4	59.6	1.0E-07	0.000	0.000	19.80
4	44.1	63.4 (U_{Lines})	1.1E-07	0.000	0.000	34.35
5	67.6	97.2	2.5E-08	0.000	0.000	15.76
6	88.7	127.5	2.0E-08	0.000	0.000	30.47
7	416.3	598.6	8.6E-10	0.000	0.000	36.09
8	418.7	602.1	2.7E-09	0.000	0.000	36.08

S7. Coordinates based on DFT optimization

B3LYP/6-31G(d)~CEP-4G

Spin multiplicity = 1

N	6.41772900	7.44413300	16.32402800
V	7.86079500	7.54836300	15.28434500
Dy	5.13594100	9.21335600	15.82834400
C	6.71609400	7.57813900	19.85945900
C	8.03669600	7.57572200	19.32412100
C	8.52720400	8.80408200	18.77863000
C	5.87331500	8.73575500	19.76824200
C	6.32325300	9.93005700	19.13066000
C	7.67875100	9.95680600	18.67832200
C	4.50533900	8.29814800	19.64366300
C	3.56668000	9.04684700	18.87005200
C	3.98545800	10.30820100	18.30986400
C	5.35780700	10.72896700	18.42925900
C	2.56696800	8.30647200	18.17526700
C	2.08298600	8.76677600	16.90386200
C	3.45885800	10.83196000	17.07195600
C	2.54098700	10.00413700	16.31149400
C	1.78288700	7.58558000	16.12226800
C	2.00188600	7.58947500	14.72402700
C	2.65927500	10.02876200	14.87964700
C	2.41037600	8.82671200	14.11910200
C	3.30953600	8.81913200	13.00319900
C	3.79871800	7.59198000	12.45447300
C	4.11722300	10.01236000	13.05865700
C	3.71824900	10.78827100	14.20649400
C	4.68534500	11.57904000	14.96387300
C	4.53171700	11.60097900	16.40690900
C	5.70037900	11.51687800	17.27429600
C	8.04088700	10.70293900	17.49836700
C	7.05851300	11.42592500	16.75237800
C	6.06839500	11.51907300	14.45083700
C	7.21598000	11.47597100	15.33742800
C	6.43186900	10.76364400	13.28338400
C	8.28864400	10.75499400	14.68891100
C	9.12090400	9.99364900	16.85884400
C	9.43270500	8.81158100	17.66378400
C	9.84401300	7.58053100	17.06161300
C	10.07234400	7.58035200	15.62781700
C	9.74827500	8.75782500	14.83292400
C	9.23114100	9.97761800	15.44675300

Spin multiplicity = 3

N	6.53525100	7.67959500	15.75044800
V	8.16216500	7.19273500	15.69180700
Dy	5.31302500	9.46973000	15.96152300
C	6.72114200	7.58410300	19.89102700
C	8.03121000	7.59051800	19.34344600
C	8.52607900	8.81565900	18.78677700
C	5.87756500	8.74583300	19.80025900
C	6.33474800	9.93493600	19.16447400
C	7.68219400	9.97621700	18.70696700
C	4.51928500	8.29475100	19.65877700
C	3.58062800	9.02407200	18.88129800
C	4.00950300	10.28018500	18.30173500
C	5.36462300	10.72789100	18.43986600
C	2.58860500	8.27982400	18.18392800
C	2.11281300	8.73155100	16.90448200
C	3.47257700	10.81524200	17.06859400
C	2.57290000	9.95527600	16.31520800
C	1.81486200	7.55675600	16.11808100
C	2.00440200	7.55606800	14.71279000
C	2.69621000	9.96975800	14.89006500
C	2.42130400	8.79005300	14.10995900
C	3.30185200	8.79403700	12.98151700
C	3.78221700	7.56436400	12.42160400
C	4.12314000	9.96776600	13.05337400
C	3.74932900	10.73303000	14.23258000
C	4.68285000	11.57571300	14.96141200
C	4.50562000	11.65468100	16.42802200
C	5.69749800	11.55299200	17.29688000
C	8.03910700	10.72368200	17.52697400
C	7.05616900	11.45622000	16.77697900
C	6.04672300	11.50192200	14.46919500
C	7.20630600	11.47674200	15.35455600
C	6.41951800	10.73201500	13.28814700
C	8.26562400	10.73631100	14.70777200
C	9.11426600	10.01713700	16.88087000
C	9.41934700	8.83172300	17.66135200
C	9.89176700	7.60575900	17.05700800
C	10.20787500	7.57869300	15.62028500
C	9.68731100	8.72983800	14.87719300
C	9.19734600	9.97083300	15.46787100

C	9.23857500	8.30930600	13.56220400	C	9.14443100	8.28228200	13.62437700
C	8.22081600	9.05653400	12.85187800	C	8.18732700	9.03842200	12.87231300
C	7.80172800	10.31486100	13.41290000	C	7.76952700	10.28331300	13.43493700
C	5.47694800	9.96440900	12.57376400	C	5.46984500	9.93016800	12.57785300
C	5.93330200	8.76623200	11.94879800	C	5.91987600	8.73879800	11.93312600
C	7.29687700	8.31883700	12.06869100	C	7.27417400	8.29384100	12.08661100
C	5.10266100	7.58967800	11.87847600	C	5.08248400	7.56656800	11.85542300
C	8.52929600	6.34834500	18.78264200	C	8.51292900	6.36819200	18.78181700
C	5.87558200	6.41627800	19.77120400	C	5.87885400	6.41631800	19.79121500
C	6.33077100	5.22732800	19.13925000	C	6.32695200	5.22767500	19.15096000
C	7.69184700	5.18534800	18.70018800	C	7.67626100	5.20337000	18.68795800
C	4.50642300	6.86061500	19.63365600	C	4.51950300	6.85149100	19.65845300
C	3.56664200	6.11893700	18.87356200	C	3.57736500	6.10412400	18.88605500
C	4.00097400	4.86015600	18.30262400	C	3.98841900	4.84353100	18.31992200
C	5.36192200	4.42566600	18.43613900	C	5.35912200	4.43766600	18.43497000
C	2.55201000	6.86293700	18.18303500	C	2.58185200	6.83968300	18.18444800
C	2.08244400	6.40775300	16.90885300	C	2.09486300	6.37905300	16.90479300
C	3.46493800	4.31087400	17.07799900	C	3.44728900	4.31322500	17.07693100
C	2.54152900	5.17088300	16.31611100	C	2.51800300	5.12920800	16.31552500
C	2.69171200	5.17069500	14.89210300	C	2.64171200	5.11033100	14.87297800
C	2.41587000	6.36008500	14.11711800	C	2.40557900	6.32088300	14.10869700
C	3.30974000	6.36494400	13.00341500	C	3.30001000	6.33504500	12.98530200
C	4.13072200	5.17984500	13.07623100	C	4.11275600	5.15611800	13.04557100
C	3.73550200	4.40657200	14.22357800	C	3.71221400	4.37119200	14.20462300
C	4.67337200	3.53393300	14.96346800	C	4.69758000	3.62382600	14.96140400
C	4.51201900	3.50049200	16.43065000	C	4.52513300	3.56823300	16.40909400
C	5.69725400	3.60111700	17.29010600	C	5.70239700	3.67192000	17.26447200
C	8.04712800	4.44417600	17.50601300	C	8.01700700	4.48440500	17.49665300
C	7.07229600	3.70695300	16.76497800	C	7.04193400	3.75203700	16.74853600
C	6.06843700	3.64916600	14.45973200	C	6.05668700	3.67664000	14.45964300
C	7.22033000	3.68584200	15.34150500	C	7.20758200	3.71043400	15.33266700
C	6.43244600	4.41312400	13.29390500	C	6.42543500	4.40603600	13.26228700
C	8.29346600	4.41192100	14.69066700	C	8.28349300	4.41307700	14.66992500
C	9.12504200	5.16056700	16.86405100	C	9.07186400	5.21129500	16.83279200
C	9.43350500	6.33827900	17.66908500	C	9.38680700	6.38591100	17.63970900
C	9.75840900	6.40296400	14.83000500	C	9.87781400	6.36804900	14.80031200
C	9.24340100	5.17621700	15.44813400	C	9.25249000	5.18659800	15.40972300
C	9.24512300	6.85326100	13.55913000	C	9.19408200	6.84486700	13.60092600
C	8.22260200	6.11141900	12.84844700	C	8.20399800	6.09706900	12.84649500
C	7.80279200	4.85252100	13.41528800	C	7.78624800	4.84287000	13.38687100
C	5.48010100	5.21291600	12.57179000	C	5.46498000	5.20253800	12.56710600
C	5.93455900	6.40951800	11.94877400	C	5.91845800	6.40029500	11.93360100
C	7.29882700	6.85267200	12.06674100	C	7.27999100	6.84650100	12.07966900
Dy	5.25571000	5.72962900	15.98108800	Dy	4.94949000	5.99768900	15.84368600

Spin multiplicity = 5

N	6.43367700	7.48157300	16.35684600
V	7.86396200	7.55487800	15.29942200
Dy	5.12777400	9.23049100	15.87959700
C	6.72004500	7.57038700	19.86848600
C	8.03573800	7.56992800	19.33495700
C	8.52350500	8.79507000	18.78561900
C	5.87424300	8.74397700	19.77787700
C	6.32612700	9.94342700	19.14928500
C	7.66755700	9.96410400	18.68780100
C	4.52235500	8.30902200	19.64390900
C	3.58898700	9.05243200	18.86911800
C	3.99702800	10.31942200	18.31370100
C	5.35725300	10.74325700	18.44098600
C	2.59224400	8.30858800	18.16479200
C	2.10943700	8.76149100	16.89525100
C	3.46253700	10.84457400	17.06333700
C	2.55658500	10.01211200	16.30053700
C	1.81765400	7.58787300	16.10990200
C	2.01889300	7.59189700	14.70610500
C	2.66958700	10.03480100	14.87069400
C	2.41910400	8.82769500	14.09897000
C	3.30089300	8.82021900	12.97779700
C	3.78332400	7.58648600	12.41641100
C	4.11724000	10.00846500	13.03982000
C	3.72363500	10.78752000	14.20046400
C	4.69716600	11.56263400	14.95305300
C	4.52673900	11.61558200	16.40822500
C	5.70273200	11.52435400	17.27430800
C	8.02939600	10.70123000	17.50867600
C	7.04334900	11.43412300	16.75873000
C	6.05492300	11.50810900	14.45670000
C	7.20826600	11.48013100	15.34020700
C	6.42507000	10.75663200	13.26843900
C	8.26925000	10.75299100	14.69706100
C	9.09780100	9.99056500	16.86725200
C	9.40707300	8.80020600	17.67150600
C	9.82922500	7.57132000	17.06910600
C	10.06055000	7.57452200	15.63025100
C	9.73377200	8.75232000	14.83703600
C	9.20017700	9.96672500	15.45102300
C	9.22200300	8.30292600	13.56404100
C	8.20217000	9.05687600	12.85624500

Spin multiplicity = 7

N	6.25981500	7.55032400	16.11613900
V	7.87314100	7.55017900	15.25499200
Dy	5.29860500	9.41756900	16.06867700
C	6.72189300	7.59125700	19.89231700
C	8.03813500	7.58797600	19.34689300
C	8.52964000	8.81052900	18.79621000
C	5.88384200	8.74925000	19.78908300
C	6.33819800	9.94790100	19.15853800
C	7.67987000	9.97241700	18.69785300
C	4.51218900	8.30784100	19.65665300
C	3.58278000	9.04333400	18.88141400
C	4.00713200	10.30875000	18.30759500
C	5.36761800	10.75162400	18.44316900
C	2.57835400	8.30291800	18.18563300
C	2.09783200	8.75421000	16.90851200
C	3.47117300	10.83992100	17.07185800
C	2.56632400	9.97707700	16.31313800
C	1.80663200	7.58053100	16.12150600
C	1.99520300	7.57614600	14.71654800
C	2.69676700	9.98521800	14.89336000
C	2.41459900	8.80187800	14.10652300
C	3.28720300	8.79995400	12.97713800
C	3.77082300	7.57221500	12.42176800
C	4.12311700	9.97150200	13.05635400
C	3.75188500	10.73755200	14.23182900
C	4.69533200	11.56004000	14.96112400
C	4.51320500	11.65910500	16.42485700
C	5.70459600	11.56845800	17.29572600
C	8.04020700	10.71593500	17.50986500
C	7.06051500	11.45528800	16.76800700
C	6.05197800	11.49057800	14.46226500
C	7.21377800	11.47160900	15.34316100
C	6.42093900	10.73355500	13.27845200
C	8.27112500	10.74290200	14.69254100
C	9.10578400	9.99764300	16.86504100
C	9.40647600	8.81070400	17.66861800
C	9.83236400	7.58217800	17.07076400
C	10.05890600	7.57633200	15.63585900
C	9.73453500	8.75217100	14.83948600
C	9.21086900	9.96500300	15.45073600
C	9.22176000	8.29655200	13.56294700
C	8.20321800	9.04584600	12.84780200

C	7.78103600	10.30677600	13.40653900	C	7.78132100	10.29415200	13.40932400
C	5.46616400	9.96293500	12.57238800	C	5.46637800	9.93585700	12.57706400
C	5.92445200	8.75574100	11.94183500	C	5.91861100	8.73546600	11.94082000
C	7.27429500	8.31321000	12.07340000	C	7.28012200	8.29562200	12.06713100
C	5.08554600	7.58439200	11.86550900	C	5.07638200	7.56978300	11.86162200
C	8.52882600	6.34305300	18.78861600	C	8.52764800	6.36343500	18.79959800
C	5.88318200	6.40786200	19.78363900	C	5.87800400	6.42975000	19.79163000
C	6.34129000	5.20429700	19.15803900	C	6.32914100	5.23349500	19.16066200
C	7.68172500	5.17610100	18.69924100	C	7.67177500	5.20097700	18.70150700
C	4.52498600	6.85349500	19.63997800	C	4.50993500	6.86807900	19.66269700
C	3.59171800	6.11966600	18.86829300	C	3.57565300	6.12941800	18.88927400
C	4.01040200	4.84669600	18.30584000	C	3.99105000	4.86288800	18.31754100
C	5.36868100	4.40156600	18.44563000	C	5.35715000	4.44309500	18.44102500
C	2.59593100	6.86561000	18.16668600	C	2.57777600	6.86682600	18.18882500
C	2.11363000	6.41264600	16.89643700	C	2.09881000	6.40548300	16.90924700
C	3.47326500	4.31610000	17.06623800	C	3.44103000	4.30854400	17.09150600
C	2.57849400	5.18139000	16.30551300	C	2.52989400	5.16048800	16.32435000
C	2.70694700	5.17288800	14.88421200	C	2.66231900	5.14286100	14.89452100
C	2.42874700	6.35848600	14.09800000	C	2.41043700	6.34405000	14.11633600
C	3.29984800	6.35592400	12.97218100	C	3.28959500	6.34619000	12.98959900
C	4.13161100	5.17752400	13.04747200	C	4.11345100	5.16988100	13.06564900
C	3.75962000	4.41850600	14.22280900	C	3.72071000	4.38774600	14.22971300
C	4.70493500	3.59814400	14.95835200	C	4.68276000	3.58955300	14.97163000
C	4.51979900	3.50267800	16.41877200	C	4.50386600	3.51653200	16.43608300
C	5.70807800	3.59041300	17.29141500	C	5.69488300	3.64696500	17.28863400
C	8.04389600	4.43711800	17.51203000	C	8.03411600	4.45363700	17.52243700
C	7.06169300	3.69780700	16.76612100	C	7.05102200	3.73291000	16.77446500
C	6.05907400	3.66090900	14.46097500	C	6.04155000	3.67687300	14.48261900
C	7.22033900	3.67489000	15.34202700	C	7.20707600	3.69375000	15.35652200
C	6.43009900	4.41226600	13.27193200	C	6.41592100	4.41683800	13.28924100
C	8.28163100	4.39723400	14.69187400	C	8.26772800	4.40719400	14.70172400
C	9.11304800	5.14990600	16.86752300	C	9.10068100	5.16694000	16.87372900
C	9.41315200	6.33837600	17.66957000	C	9.40532900	6.35625800	17.67616400
C	9.74206200	6.39699100	14.83335000	C	9.74444200	6.39512300	14.84035600
C	9.21350400	5.17967000	15.44966000	C	9.20929700	5.18852800	15.45964900
C	9.22591400	6.85083800	13.56040200	C	9.22767000	6.84636900	13.56239700
C	8.20551000	6.10223200	12.85075300	C	8.20224000	6.09727700	12.85099900
C	7.78761600	4.84885200	13.40526100	C	7.77840400	4.84996700	13.41590500
C	5.47407700	5.20915400	12.56933800	C	5.46048700	5.20870900	12.58726400
C	5.92798600	6.40866500	11.93457500	C	5.91404000	6.40690500	11.94643300
C	7.27722600	6.84820900	12.07080300	C	7.27959300	6.84538900	12.07061200
Dy	5.30069100	5.76485000	16.08876200	Dy	5.06672300	5.81122600	15.96847900

Spin multiplicity = 9

Spin multiplicity = 11

N	6.31351300	7.58652100	15.94791000	N	6.32069400	7.61024300	15.90016100
V	8.05477400	7.58853800	15.48358500	V	8.04647600	7.63165700	15.44029900
Dy	5.14561600	9.38848900	15.96170100	Dy	5.03075000	9.32650500	15.95202700
C	6.71892100	7.57471300	19.88473200	C	6.71847200	7.55679200	19.88437500
C	8.02534200	7.57494900	19.33030300	C	8.02538400	7.55229400	19.32970300
C	8.51289300	8.80196700	18.77805200	C	8.51453500	8.77715100	18.77648900
C	5.87892800	8.74108300	19.79171500	C	5.88321300	8.72653700	19.79160000
C	6.33438900	9.93251100	19.15638800	C	6.34181700	9.91520700	19.15446700
C	7.68045400	9.97067400	18.69929400	C	7.68739100	9.94939300	18.69699500
C	4.52115100	8.29839200	19.65451900	C	4.52294300	8.29140500	19.65604700
C	3.57917600	9.03468400	18.88236200	C	3.58233300	9.03270900	18.88452100
C	4.00065600	10.29463800	18.30415200	C	4.00285800	10.29364000	18.30889600
C	5.36449700	10.72179100	18.43139700	C	5.37302900	10.70302600	18.42806700
C	2.58310200	8.29603200	18.18700600	C	2.58495100	8.29914300	18.18680600
C	2.11266100	8.75085200	16.90226100	C	2.11350700	8.76101200	16.90155600
C	3.45441300	10.84325900	17.07451600	C	3.45228800	10.85105200	17.08158500
C	2.55322100	9.98677100	16.31347000	C	2.53720200	10.00698700	16.31761800
C	1.82231000	7.57720200	16.11374900	C	1.81621800	7.58556500	16.11442800
C	2.00654000	7.57758900	14.70849300	C	2.00086700	7.58545000	14.70873400
C	2.68267300	9.99817700	14.88216700	C	2.67346800	10.01621000	14.88194400
C	2.42092400	8.80860700	14.10560300	C	2.42205500	8.81763100	14.11014200
C	3.30528300	8.80559300	12.97846000	C	3.31078500	8.80823500	12.98592900
C	3.78573900	7.57681400	12.41426800	C	3.78425500	7.57838000	12.41627400
C	4.12666400	9.97809400	13.04989800	C	4.13446700	9.97871400	13.05536200
C	3.74197900	10.75329500	14.21975300	C	3.74352600	10.76179000	14.21742800
C	4.69083000	11.56857600	14.95869400	C	4.70782700	11.55375400	14.96101700
C	4.50984400	11.64839800	16.42061800	C	4.52595500	11.62912000	16.42063900
C	5.70153800	11.52429800	17.27923800	C	5.71489700	11.49029600	17.27070500
C	8.03898100	10.71134600	17.51715500	C	8.04850400	10.68719100	17.51387700
C	7.05790100	11.43355300	16.76485600	C	7.07145900	11.40891000	16.76038300
C	6.05381300	11.47969800	14.46965900	C	6.06844000	11.45846700	14.47363600
C	7.21416300	11.45814900	15.34591400	C	7.23052500	11.44008700	15.34520000
C	6.42772600	10.73446800	13.27670400	C	6.44092100	10.72418900	13.27600300
C	8.28339400	10.74625200	14.68866400	C	8.29997100	10.73378400	14.68455900
C	9.10518000	9.99793400	16.86160700	C	9.10780600	9.96942000	16.85497400
C	9.37912900	8.80293600	17.63833800	C	9.37592400	8.77070000	17.63331000
C	9.82985000	7.57468400	17.03539800	C	9.82938100	7.54471600	17.03643500
C	10.15884600	7.57612400	15.61741900	C	10.15254300	7.55383500	15.61700500
C	9.78254200	8.76620700	14.83114600	C	9.80497800	8.75019700	14.82410500
C	9.22517100	9.97353100	15.44363200	C	9.24065400	9.95614800	15.43778600
C	9.19866800	8.30126600	13.59129600	C	9.21532600	8.28764300	13.58250500
C	8.20626200	9.04989400	12.85132600	C	8.21641000	9.03838300	12.84817500
C	7.78594100	10.29711700	13.41170600	C	7.80042300	10.28784800	13.40593200
C	5.47612100	9.93747000	12.57180800	C	5.48465000	9.93320700	12.57429300

C	5.92741200	8.74494200	11.92955700
C	7.28664300	8.30158000	12.07234700
C	5.08931600	7.57693900	11.85379200
C	8.51266000	6.34763700	18.77795800
C	5.87803200	6.40808100	19.79297600
C	6.33299200	5.21520300	19.15985100
C	7.67918500	5.17956100	18.70005200
C	4.52058600	6.85265400	19.65438300
C	3.57812500	6.11653600	18.88307300
C	4.00021000	4.85447600	18.30826000
C	5.36252300	4.41925900	18.43934100
C	2.58152900	6.85708400	18.18769900
C	2.11018000	6.40506000	16.90413000
C	3.45986800	4.31490000	17.07316700
C	2.56406300	5.17627600	16.31338700
C	2.69392700	5.16804900	14.88537500
C	2.42008900	6.34847600	14.10322600
C	3.30212800	6.34870000	12.97364800
C	4.12707000	5.17864800	13.04803200
C	3.75206700	4.41396000	14.22596200
C	4.69224500	3.59098800	14.96078600
C	4.50856600	3.50054200	16.42277900
C	5.69949600	3.60840600	17.28840300
C	8.03611000	4.43909100	17.51690200
C	7.05595500	3.70924400	16.76774000
C	6.05390100	3.66873700	14.46761700
C	7.21166600	3.69103800	15.34667100
C	6.42597000	4.41853800	13.27760700
C	8.28043300	4.40814000	14.69037500
C	9.10432800	5.15238000	16.86231000
C	9.38028600	6.34572100	17.63929300
C	9.77573000	6.38812400	14.83322400
C	9.22235100	5.17895800	15.44473200
C	9.19583500	6.85267000	13.59248800
C	8.20466300	6.10400800	12.85220400
C	7.78336100	4.85766100	13.41406700
C	5.47502000	5.21673700	12.57137400
C	5.92720000	6.40903700	11.92882900
C	7.28623500	6.85222100	12.07218400
Dy	5.23009400	5.74160500	16.01880000

Spin multiplicity = 13

N	6.31359400	7.57785100	15.91543100
V	8.05740100	7.57777800	15.47422800

C	5.93132100	8.73892300	11.93319600
C	7.28988200	8.29196200	12.07322600
C	5.08869500	7.57467400	11.85650800
C	8.51063700	6.32403300	18.77739300
C	5.87329100	6.39389500	19.79316700
C	6.32539000	5.19843900	19.16206500
C	7.67129700	5.15987300	18.69977200
C	4.51756900	6.84669800	19.65244100
C	3.57379100	6.11690800	18.87923100
C	3.99832300	4.85748000	18.30296400
C	5.35348600	4.39804500	18.44536800
C	2.57553700	6.86060100	18.18704600
C	2.09830700	6.41384800	16.90579200
C	3.46554500	4.33454900	17.06314900
C	2.56548200	5.19541200	16.30975000
C	2.68989900	5.18471400	14.88690800
C	2.40812900	6.35627000	14.09933300
C	3.29227800	6.35279100	12.97022500
C	4.11871800	5.18404100	13.04967600
C	3.75034800	4.43058500	14.23495000
C	4.67831000	3.59230800	14.96272500
C	4.49536200	3.49583100	16.42354800
C	5.68635800	3.56986000	17.30163500
C	8.02512200	4.41593200	17.51660000
C	7.04510500	3.67356900	16.77406800
C	6.04083900	3.65172100	14.46572900
C	7.19799100	3.66671300	15.34885000
C	6.41282500	4.41218500	13.28268800
C	8.26572000	4.39543900	14.69633300
C	9.10122900	5.12486700	16.86453800
C	9.38236400	6.31673000	17.64136800
C	9.75878200	6.37131700	14.83829600
C	9.21429500	5.15690600	15.44856200
C	9.19284700	6.83825600	13.59232800
C	8.19777800	6.09235100	12.85424700
C	7.76969700	4.85067100	13.42149900
C	5.46525600	5.21535500	12.57436800
C	5.92230400	6.40420400	11.93027700
C	7.28311800	6.84270800	12.07351700
Dy	5.36645700	5.68971000	16.06020100

Spin multiplicity = 15

N	6.27709500	7.63920200	16.14576800
V	7.87864600	7.61934800	15.25945900

Dy	5.15828800	9.38552900	16.00619400	Dy	5.03935600	9.32200800	15.89761200
C	6.71935700	7.57615000	19.88479700	C	6.72345900	7.56700400	19.88575100
C	8.02611000	7.57616200	19.33008800	C	8.04083100	7.56841900	19.34315500
C	8.51343600	8.80311000	18.77779800	C	8.53263600	8.79085400	18.79280100
C	5.87918100	8.74254700	19.79155600	C	5.87913100	8.73149700	19.78121900
C	6.33447500	9.93483500	19.15763200	C	6.33323400	9.92383200	19.14626800
C	7.68075500	9.97212900	18.69975700	C	7.67615900	9.95514700	18.68944200
C	4.52152400	8.29918000	19.65284200	C	4.51238800	8.29690900	19.65533400
C	3.57887500	9.03556900	18.88210300	C	3.57803200	9.03511400	18.87749100
C	3.99863100	10.29847100	18.30715200	C	3.99664600	10.29543000	18.30064100
C	5.36330900	10.72661200	18.43457200	C	5.36412800	10.70885300	18.42121100
C	2.58279000	8.29563500	18.18640300	C	2.57991600	8.30008600	18.17795100
C	2.11192400	8.74970000	16.90205800	C	2.10093900	8.76135900	16.89656800
C	3.45354900	10.84485900	17.07541800	C	3.44857100	10.84813800	17.07351500
C	2.55471700	9.98512000	16.31344600	C	2.53008300	10.00584700	16.30916700
C	1.82278500	7.57638400	16.11267700	C	1.80979400	7.58465000	16.11073900
C	2.00438500	7.57631700	14.70666300	C	2.00243500	7.58692900	14.70634800
C	2.68625600	9.99264400	14.88374800	C	2.66291500	10.02649700	14.87600100
C	2.41851800	8.80666900	14.10341300	C	2.42082400	8.81861200	14.10567500
C	3.30332500	8.80443500	12.97656200	C	3.30510200	8.81081900	12.98155100
C	3.78586900	7.57624500	12.41449700	C	3.78316100	7.58321400	12.41314500
C	4.12783600	9.97502000	13.05087100	C	4.12632200	9.98803400	13.05266900
C	3.74718700	10.74457700	14.22341300	C	3.72538600	10.78054100	14.20872400
C	4.69387300	11.56022400	14.96224500	C	4.69481500	11.57001600	14.95195000
C	4.50937700	11.65038000	16.42255700	C	4.51791900	11.62889700	16.41446500
C	5.70079100	11.53130700	17.28259800	C	5.70568200	11.49520000	17.26620500
C	8.03939100	10.71285400	17.51763800	C	8.04205200	10.69912100	17.51190300
C	7.05761700	11.43610500	16.76616600	C	7.06219200	11.41778900	16.75853900
C	6.05470100	11.47459200	14.47198300	C	6.05640500	11.47973200	14.46686700
C	7.21450800	11.45696900	15.34737600	C	7.22107900	11.46167700	15.34233800
C	6.42822000	10.73150700	13.27833000	C	6.43196200	10.73654000	13.27673300
C	8.28397800	10.74535400	14.68949700	C	8.28205800	10.74503300	14.69135300
C	9.10598000	9.99937800	16.86211700	C	9.10947900	9.98286000	16.86660900
C	9.38060300	8.80451100	17.63848200	C	9.41337600	8.79571300	17.67364100
C	9.83128800	7.57614700	17.03500200	C	9.83901700	7.56718900	17.07072700
C	10.16124500	7.57626000	15.61684800	C	10.06575600	7.57225700	15.63579900
C	9.78273700	8.76560900	14.83132500	C	9.75639800	8.75308500	14.83679800
C	9.22555400	9.97336500	15.44394500	C	9.22115900	9.96216200	15.45307900
C	9.20170300	8.30079300	13.59005000	C	9.23896000	8.30088500	13.55954100
C	8.20770400	9.04900300	12.85136900	C	8.21547600	9.05059700	12.84521600
C	7.78644200	10.29533700	13.41285100	C	7.79415800	10.30109200	13.40609200
C	5.47681400	9.93549100	12.57289400	C	5.47546000	9.94443100	12.57618600
C	5.92832300	8.74371700	11.93002400	C	5.92751400	8.74436200	11.93792000
C	7.28770600	8.30073700	12.07265500	C	7.29280800	8.30321700	12.06554800

C	5.09003100	7.57626800	11.85449900
C	8.51346900	6.34919700	18.77782700
C	5.87914800	6.40976100	19.79169600
C	6.33448900	5.21728500	19.15806100
C	7.68074700	5.18015300	18.69994800
C	4.52154900	6.85334700	19.65279200
C	3.57898300	6.11705900	18.88199600
C	3.99923800	4.85446800	18.30698700
C	5.36343300	4.42505700	18.43529900
C	2.58271800	6.85697800	18.18641500
C	2.11184800	6.40322200	16.90211100
C	3.45485500	4.30899100	17.07472000
C	2.55591700	5.16865600	16.31308300
C	2.68677800	5.16070000	14.88365600
C	2.41827800	6.34606200	14.10308000
C	3.30298200	6.34805900	12.97607000
C	4.12753200	5.17746400	13.05043400
C	3.74744700	4.40853300	14.22355000
C	4.69359500	3.59228000	14.96223300
C	4.50919300	3.50169900	16.42266500
C	5.70074100	3.61976800	17.28339600
C	8.03922600	4.43939000	17.51781200
C	7.05750000	3.71552200	16.76658800
C	6.05451400	3.67701400	14.47168400
C	7.21417800	3.69508900	15.34749900
C	6.42789200	4.42086800	13.27846700
C	8.28351400	4.40728600	14.68978400
C	9.10589900	5.15296600	16.86228300
C	9.38073400	6.34770400	17.63860900
C	9.78190300	6.38708900	14.83162400
C	9.22513000	5.17920500	15.44419300
C	9.20135000	6.85180600	13.59022700
C	8.20747500	6.10356400	12.85153600
C	7.78602000	4.85736300	13.41323700
C	5.47650900	5.21699800	12.57282000
C	5.92822900	6.40878100	11.93000700
C	7.28762600	6.85175100	12.07268300
Dy	5.16647000	5.76442600	16.00669300

Spin multiplicity = 17

N	6.47055900	7.60628600	16.40385100
V	7.89027900	7.58550900	15.31887600
Dy	5.12055500	9.19903900	15.94611800
C	6.72194500	7.57302300	19.87176900

C	5.08907700	7.58322800	11.85470800
C	8.53172100	6.34365600	18.79673200
C	5.88549600	6.41152600	19.78808900
C	6.33963200	5.20837700	19.16307000
C	7.68157100	5.18316900	18.70232400
C	4.51366100	6.85749700	19.65156700
C	3.58674200	6.12403500	18.87451800
C	4.01619200	4.85942900	18.30351000
C	5.36935200	4.39783800	18.45297900
C	2.58113400	6.86381700	18.17650000
C	2.10081000	6.41320700	16.90090400
C	3.48937400	4.33974000	17.06212700
C	2.58147200	5.19798900	16.30454500
C	2.70788400	5.18422500	14.88822000
C	2.42133800	6.36042300	14.09631500
C	3.29516500	6.35782900	12.96787900
C	4.13207900	5.18641800	13.05201000
C	3.76438200	4.43068800	14.23332200
C	4.69979800	3.60467000	14.96254900
C	4.51725700	3.50447500	16.42570000
C	5.70587000	3.57062600	17.30813900
C	8.03929000	4.43803100	17.51279000
C	7.06237200	3.68544200	16.77619600
C	6.05617200	3.65822500	14.46138100
C	7.21518400	3.67575600	15.34724300
C	6.42627800	4.41701600	13.27929800
C	8.27401300	4.40734200	14.69692200
C	9.10800200	5.15377100	16.86805600
C	9.41021200	6.34068400	17.66898900
C	9.73600200	6.39668500	14.84219200
C	9.21411300	5.18421000	15.45347300
C	9.22631900	6.85109600	13.56469300
C	8.21112300	6.10183600	12.84842000
C	7.78634100	4.85598200	13.41264000
C	5.47447400	5.21646100	12.57393100
C	5.92999300	6.41454100	11.93588300
C	7.29029100	6.85257300	12.06450500
Dy	5.41866800	5.71228300	16.09749500

Spin multiplicity = 19

N	6.48063700	7.57554300	16.45026200
V	7.87261900	7.57675200	15.34150500
Dy	5.15825600	9.22535700	15.97109000
C	6.73479900	7.57360000	19.88180700

C	8.04519400	7.57318800	19.34068500	C	8.06247100	7.57461000	19.35666600
C	8.53681700	8.79608200	18.79201400	C	8.54527100	8.79712200	18.79710200
C	5.87968800	8.73583900	19.78264200	C	5.89098700	8.73091900	19.78307600
C	6.33956400	9.93085700	19.15369300	C	6.34578600	9.92218200	19.15597100
C	7.68203700	9.95788400	18.69358200	C	7.69340300	9.95332100	18.69798400
C	4.51521000	8.29650200	19.65167300	C	4.51649000	8.28532900	19.64476200
C	3.57918900	9.02448700	18.87192900	C	3.57103500	9.02146200	18.86565700
C	4.00938500	10.28077500	18.30027900	C	4.00507400	10.27218600	18.29837300
C	5.36903600	10.71778000	18.43727000	C	5.37103400	10.70699100	18.43427100
C	2.56096900	8.29254900	18.17981000	C	2.54384500	8.28680400	18.18896400
C	2.08081300	8.74511400	16.89991200	C	2.07502800	8.74061100	16.89468600
C	3.47226000	10.80774800	17.05915000	C	3.47355400	10.80414800	17.06140500
C	2.55096500	9.97457300	16.30268500	C	2.54328200	9.96304100	16.30249100
C	1.77709300	7.57622700	16.11045700	C	1.75863200	7.57311400	16.10884500
C	1.98028600	7.57701100	14.69891200	C	1.96087000	7.57514300	14.68892400
C	2.67763800	9.99434600	14.87522200	C	2.68013600	9.97337300	14.87010800
C	2.40344200	8.80573500	14.09001900	C	2.38658600	8.79668300	14.08393600
C	3.28477500	8.80362500	12.96437000	C	3.28057900	8.79493000	12.95969600
C	3.76740400	7.57856700	12.40054300	C	3.76034800	7.57504900	12.39194600
C	4.12345200	9.97647500	13.04467300	C	4.12047800	9.96800000	13.04180000
C	3.74387500	10.73745300	14.21410000	C	3.74282700	10.71843500	14.21103100
C	4.71591100	11.51917100	14.95676200	C	4.70229500	11.52914800	14.95696200
C	4.53845700	11.57815000	16.40660000	C	4.53313300	11.57162900	16.40894100
C	5.71465700	11.49030100	17.27075200	C	5.71211500	11.47924800	17.27353000
C	8.04597300	10.69488200	17.50806400	C	8.05147600	10.69067200	17.50745400
C	7.06205900	11.42057000	16.75552300	C	7.07073100	11.41525000	16.75628000
C	6.06587400	11.47967500	14.45760500	C	6.07350900	11.46736600	14.45145500
C	7.22677200	11.46922400	15.33816000	C	7.23165600	11.45924400	15.33454000
C	6.43292500	10.74284800	13.26502100	C	6.43140900	10.73840300	13.27236200
C	8.28711900	10.75186500	14.69048300	C	8.29502700	10.74597800	14.69006200
C	9.11794900	9.99133000	16.86644200	C	9.12696500	9.98677500	16.86929500
C	9.42316100	8.80170400	17.67249000	C	9.43156100	8.80141000	17.67081700
C	9.84726000	7.57469200	17.07127300	C	9.86465100	7.57547200	17.07281200
C	10.07738200	7.57590200	15.62783900	C	10.08130800	7.57511400	15.62861500
C	9.75120100	8.75318400	14.83284100	C	9.75325100	8.74973200	14.83480800
C	9.22019700	9.96822900	15.44912700	C	9.22616100	9.96181300	15.44753900
C	9.23091900	8.30088900	13.55983100	C	9.23126900	8.29701000	13.56127600
C	8.20934500	9.05096900	12.84884000	C	8.21153200	9.04603300	12.85384500
C	7.79171200	10.30139400	13.40253500	C	7.79499100	10.29177600	13.40721800
C	5.47168700	9.94740600	12.56929200	C	5.46478900	9.94787800	12.54410400
C	5.92679700	8.74958600	11.93418400	C	5.91989200	8.75285600	11.92578500
C	7.27883000	8.31053900	12.06856900	C	7.27585300	8.30347200	12.06857200
C	5.08279000	7.57805300	11.86284200	C	5.08173300	7.57711500	11.85333000
C	8.53705100	6.35058400	18.79250900	C	8.54611800	6.35329300	18.79674800

C	5.87919300	6.40942500	19.78462100	C	5.89142200	6.41588200	19.78784500
C	6.33952100	5.21396700	19.15612500	C	6.34570600	5.22580000	19.15746300
C	7.68246900	5.18801800	18.69485500	C	7.69066900	5.19807000	18.69588000
C	4.51417300	6.84937200	19.65305500	C	4.51635600	6.86068000	19.64430100
C	3.57772000	6.12264300	18.87315200	C	3.56729900	6.12227400	18.87103200
C	4.00489600	4.86051900	18.30591100	C	3.99911600	4.86479000	18.30520000
C	5.36659600	4.42111700	18.44284500	C	5.36933400	4.43717000	18.43786400
C	2.56023900	6.85741600	18.18003600	C	2.54450400	6.86163300	18.18859200
C	2.08061900	6.40757900	16.89991500	C	2.07436700	6.40904300	16.89377800
C	3.47149700	4.33965600	17.06050500	C	3.46654300	4.33561100	17.06674600
C	2.55636200	5.18177700	16.30139300	C	2.53170800	5.17494700	16.30445500
C	2.68453700	5.16943400	14.87795400	C	2.67986000	5.17579700	14.87340600
C	2.40050100	6.35181600	14.08905000	C	2.39294400	6.35165300	14.08766300
C	3.28512000	6.35432200	12.96421900	C	3.28318000	6.35439500	12.95784700
C	4.12482600	5.18280100	13.04378500	C	4.12385900	5.18383400	13.03898100
C	3.75300500	4.43213100	14.21947100	C	3.74855300	4.43765800	14.21299900
C	4.71766000	3.64630200	14.96047600	C	4.71852500	3.65550700	14.96320300
C	4.53562200	3.56705300	16.40906100	C	4.53641800	3.58603100	16.40990100
C	5.71282900	3.64338500	17.27704100	C	5.71524500	3.67465000	17.27323700
C	8.04570800	4.45131600	17.50834700	C	8.05206600	4.46225000	17.50691800
C	7.06106500	3.72265700	16.75750200	C	7.06777100	3.74412100	16.75296100
C	6.06496700	3.68194700	14.45865600	C	6.07416800	3.69395500	14.45655300
C	7.22538900	3.68454700	15.33869100	C	7.23577000	3.68969000	15.33490400
C	6.43086200	4.41473100	13.26410600	C	6.43538700	4.41513400	13.26870500
C	8.28532300	4.40239000	14.69033500	C	8.29579100	4.40410500	14.68986000
C	9.11619200	5.15743900	16.86621100	C	9.12600200	5.16474600	16.86825800
C	9.42058600	6.34653800	17.67163500	C	9.43364700	6.34619200	17.67295400
C	9.74782700	6.40000400	14.83291400	C	9.75411400	6.40145500	14.83266400
C	9.21737200	5.18487500	15.44840800	C	9.23076100	5.18454300	15.44574700
C	9.22927500	6.85343700	13.56015100	C	9.23255600	6.85398600	13.56095800
C	8.20836500	6.10459800	12.84853900	C	8.21534300	6.10421700	12.84727600
C	7.78908800	4.85543300	13.40277000	C	7.79444700	4.85872200	13.40544000
C	5.47066400	5.20897200	12.56677300	C	5.46729700	5.20825400	12.55278400
C	5.92578300	6.40703100	11.93243000	C	5.92165800	6.40478400	11.92985100
C	7.27777200	6.84645500	12.06920500	C	7.27626900	6.85029400	12.07260800
Dy	5.17768700	5.93607200	16.06357800	Dy	5.08566600	5.99173600	16.04799100

PBE0/6-31G(d)~CEP-4G

**Spin multiplicity = 9**				**Spin multiplicity = 13**			
N	6.30626100	7.60827400	15.87268400	N	6.32955500	7.59242700	15.98782600
V	8.04370300	7.61518100	15.44631300	V	8.05051000	7.59494200	15.48123100
Dy	5.05668900	9.34856100	15.98345100	Dy	5.16443900	9.37748600	16.02613700

C	6.71359700	7.57225400	19.86954000	C	6.71462200	7.57662600	19.86879600
C	8.01494800	7.57288100	19.31549700	C	8.01583400	7.57609400	19.31586100
C	8.49966500	8.79376900	18.76451700	C	8.50053100	8.79755600	18.76509600
C	5.87794300	8.73367900	19.77629100	C	5.87829400	8.73868100	19.77603700
C	6.33119000	9.91859500	19.14042100	C	6.33128500	9.92577900	19.14375800
C	7.67156000	9.95716600	18.68505400	C	7.67210100	9.96139800	18.68664700
C	4.52502500	8.29530700	19.64028300	C	4.52604700	8.29787700	19.63859400
C	3.58662600	9.03039500	18.87128600	C	3.58734100	9.03160100	18.86960200
C	4.00111700	10.28773000	18.29602500	C	4.00506100	10.28881900	18.29591700
C	5.36443900	10.69923000	18.41467400	C	5.36483200	10.71483800	18.42333000
C	2.59706500	8.29455000	18.17651600	C	2.59529500	8.29487200	18.17647600
C	2.13044900	8.75169000	16.89584200	C	2.12800100	8.74761600	16.89675300
C	3.45174700	10.84454300	17.07506300	C	3.46184200	10.83477200	17.07014600
C	2.55193300	9.99305700	16.31465100	C	2.56783400	9.97768900	16.31172600
C	1.83851800	7.58097100	16.11254500	C	1.83850800	7.57881500	16.11132300
C	2.02039600	7.58073600	14.71191600	C	2.01922900	7.57738900	14.71065400
C	2.68981100	9.99760800	14.88696700	C	2.69966000	9.98255200	14.88762100
C	2.43517000	8.80781200	14.11531700	C	2.43255900	8.80246600	14.11110400
C	3.31848700	8.80171500	12.99470600	C	3.31288600	8.79970600	12.98717300
C	3.79429800	7.57848100	12.42843700	C	3.79154900	7.57635600	12.42570700
C	4.13708200	9.96876400	13.06681400	C	4.13388200	9.96491000	13.06099600
C	3.74990200	10.74327400	14.22694000	C	3.75762300	10.72822800	14.23167300
C	4.70051400	11.54298700	14.96583900	C	4.69782600	11.53961100	14.96614900
C	4.51555000	11.62720900	16.41832000	C	4.51449800	11.63024200	16.41887300
C	5.70071400	11.49098100	17.26409500	C	5.70031200	11.51554800	17.27547300
C	8.02943600	10.69347200	17.50770700	C	8.02855000	10.69850700	17.50937300
C	7.05304000	11.40766800	16.75697400	C	7.05067600	11.41882400	16.76183800
C	6.05540200	11.45081800	14.47932200	C	6.05254300	11.45521300	14.47712300
C	7.21184300	11.43451600	15.34698900	C	7.20640100	11.43654900	15.34855500
C	6.42869300	10.71848400	13.28883400	C	6.42362300	10.71697600	13.28959600
C	8.27910500	10.73282600	14.69117900	C	8.27260100	10.72963200	14.69399100
C	9.09119300	9.98447000	16.85492100	C	9.09099400	9.98899100	16.85708300
C	9.35998600	8.79328300	17.62666300	C	9.36205500	8.79799300	17.62879800
C	9.81028700	7.57180000	17.02740400	C	9.81395400	7.57537100	17.02919100
C	10.14137200	7.57643100	15.61672200	C	10.14388500	7.57631000	15.61768200
C	9.77950100	8.76302900	14.83222200	C	9.76966200	8.76081800	14.83602200
C	9.21723200	9.96301300	15.44220200	C	9.21206900	9.96272900	15.44488300
C	9.19627800	8.29999300	13.59532500	C	9.18483400	8.29752300	13.60214900
C	8.20148300	9.04450800	12.86227900	C	8.19533200	9.04167300	12.86510000
C	7.78286100	10.28606000	13.41985700	C	7.77647000	10.28235500	13.42339100
C	5.48067200	9.92740600	12.58712200	C	5.47620200	9.92527200	12.58476100
C	5.92942200	8.74055100	11.94640500	C	5.92621800	8.73820800	11.94429600
C	7.28334300	8.29893500	12.08729900	C	7.27937100	8.29662800	12.08808700
C	5.09426200	7.57842000	11.87089400	C	5.09130600	7.57593400	11.86842100

C	8.50019900	6.35138600	18.76466800	C	8.50179600	6.35404800	18.76553800
C	5.87591800	6.41108900	19.77707500	C	5.87784300	6.41555200	19.77562800
C	6.32913100	5.22099700	19.14901600	C	6.33193800	5.22814700	19.14426100
C	7.66968600	5.18862800	18.68837700	C	7.67258700	5.19073800	18.68804900
C	4.52461800	6.85639200	19.63697800	C	4.52642000	6.85889200	19.63670100
C	3.58689900	6.12347700	18.86831200	C	3.58940200	6.12784100	18.86551900
C	4.01088600	4.86839100	18.29626600	C	4.01568800	4.87920300	18.28479700
C	5.36414900	4.42056800	18.43520200	C	5.36840200	4.44037800	18.42052100
C	2.59233600	6.86200300	18.17738100	C	2.59385700	6.86268000	18.17605300
C	2.12187200	6.41466500	16.90051500	C	2.12529200	6.41234900	16.89781300
C	3.48104000	4.34083700	17.06183600	C	3.47866200	4.33883100	17.05875300
C	2.58639000	5.19872700	16.30971900	C	2.58102000	5.19190900	16.30742700
C	2.71354700	5.19122300	14.89171200	C	2.70364500	5.17674800	14.88591200
C	2.42982200	6.35730400	14.10699100	C	2.43083800	6.35201900	14.10813500
C	3.30986600	6.35628800	12.98262300	C	3.31083200	6.35255700	12.98440300
C	4.13613900	5.19488100	13.06242300	C	4.13176800	5.18683800	13.05981100
C	3.77176100	4.44504400	14.24284900	C	3.75744500	4.42284500	14.23208800
C	4.69867400	3.61659200	14.97078100	C	4.69105300	3.59506800	14.96235100
C	4.51381000	3.51532600	16.42372800	C	4.51335500	3.51633200	16.41754600
C	5.69786800	3.59919100	17.29447800	C	5.69999900	3.62710600	17.27891800
C	8.02301200	4.45066100	17.50999300	C	8.02680900	4.45239400	17.51057600
C	7.04904400	3.71053700	16.77043300	C	7.05076900	3.72431400	16.76505700
C	6.05274000	3.68619900	14.47599600	C	6.04990700	3.67814000	14.47294500
C	7.20411500	3.70435600	15.35270600	C	7.20419100	3.70534200	15.34906900
C	6.42231600	4.43521600	13.29327800	C	6.42061100	4.43118200	13.29128200
C	8.26738400	4.42509300	14.69991100	C	8.26724300	4.42301100	14.69754600
C	9.08959600	5.16075300	16.85931100	C	9.09063200	5.16228000	16.85892600
C	9.36475800	6.34805400	17.63081100	C	9.36448300	6.35192100	17.63051700
C	9.75675200	6.39521700	14.83971400	C	9.75891500	6.39383900	14.83987700
C	9.20776100	5.18993900	15.44820900	C	9.20772000	5.18964300	15.44787600
C	9.18656100	6.85670200	13.59984900	C	9.18029800	6.85580900	13.60433600
C	8.19586600	6.11188800	12.86586400	C	8.19249000	6.11103900	12.86719200
C	7.77327800	4.87336400	13.42971700	C	7.77139800	4.87128700	13.42854900
C	5.47690800	5.23037900	12.58693600	C	5.47474500	5.22669800	12.58532000
C	5.92815600	6.41565000	11.94451100	C	5.92543900	6.41266100	11.94381600
C	7.28191700	6.85674800	12.08747800	C	7.27876000	6.85470400	12.08843300
Dy	5.33518300	5.71815400	16.07956200	Dy	5.26482000	5.73025300	15.96297400

TPSSh/6-31G(d)~CEP-4G

Spin multiplicity = 9

N	6.33013300	7.58398000	15.94475100
V	8.06906500	7.58501000	15.44822400

Spin multiplicity = 13

N	6.33194500	7.57698300	15.94942000
V	8.06446300	7.57742500	15.44938800

Dy	5.16949800	9.40016800	15.99461800	Dy	5.20606400	9.41218500	16.01438700
C	6.71718400	7.57473300	19.88517700	C	6.71727800	7.57618600	19.88529200
C	8.02309000	7.57509800	19.33024000	C	8.02342600	7.57619400	19.33041000
C	8.51100700	8.80136600	18.77784300	C	8.51101500	8.80251600	18.77772000
C	5.87713600	8.74023800	19.79074200	C	5.87688000	8.74211700	19.79145300
C	6.33298300	9.93199300	19.15617500	C	6.33284700	9.93448000	19.15766200
C	7.67841800	9.96913800	18.69880100	C	7.67847600	9.97037600	18.69927000
C	4.52110700	8.29773100	19.65136800	C	4.52070200	8.29876900	19.65139300
C	3.57875000	9.03328000	18.87919000	C	3.57796500	9.03418100	18.87920100
C	4.00098400	10.29108400	18.29915600	C	4.00155000	10.29231300	18.29988100
C	5.36451700	10.71883400	18.42725900	C	5.36411000	10.72462400	18.43058800
C	2.58196700	8.29485100	18.18510700	C	2.58003100	8.29546500	18.18596100
C	2.11369300	8.74896900	16.90161400	C	2.11165900	8.74816800	16.90249200
C	3.45499300	10.84345400	17.07242300	C	3.45782800	10.84093000	17.07102600
C	2.55789400	9.98338400	16.31304000	C	2.56214200	9.97875600	16.31255900
C	1.82350200	7.57682000	16.11284600	C	1.82304200	7.57635900	16.11296000
C	2.00458400	7.57716300	14.70681800	C	2.00394200	7.57634100	14.70700400
C	2.68920400	9.99131300	14.88437000	C	2.69257600	9.98584600	14.88513500
C	2.42069300	8.80680400	14.10464600	C	2.41954600	8.80493300	14.10345600
C	3.30379100	8.80434200	12.97732500	C	3.30229300	8.80338800	12.97560800
C	3.78282100	7.57682900	12.41072100	C	3.78296000	7.57629100	12.41097600
C	4.12875300	9.97364000	13.05280200	C	4.12851300	9.97163000	13.05213300
C	3.74956900	10.74277200	14.22615500	C	3.75298800	10.73679700	14.22861400
C	4.69048900	11.56659000	14.96184500	C	4.69040500	11.56458800	14.96267900
C	4.50773100	11.65170500	16.42128400	C	4.50691000	11.65437700	16.42215800
C	5.69947500	11.52872700	17.27936700	C	5.69889300	11.53860800	17.28379300
C	8.03546600	10.70874700	17.51739400	C	8.03502800	10.71006900	17.51751700
C	7.05487200	11.43145400	16.76520200	C	7.05430700	11.43613400	16.76695000
C	6.05219800	11.47126200	14.47301100	C	6.05193500	11.47279300	14.47257600
C	7.21180500	11.45206100	15.34648100	C	7.21086900	11.45366400	15.34715000
C	6.42567300	10.72948600	13.27965000	C	6.42470900	10.72826400	13.28081500
C	8.27973300	10.74113400	14.68880000	C	8.27850900	10.74055300	14.68984400
C	9.10167400	9.99600700	16.86378300	C	9.10164000	9.99735300	16.86405500
C	9.37499200	8.80383500	17.63789800	C	9.37501900	8.80518000	17.63787600
C	9.81853800	7.57494200	17.02871000	C	9.81858500	7.57614100	17.02901100
C	10.16396400	7.57615000	15.61559900	C	10.16220500	7.57625200	15.61572700
C	9.78573000	8.76505000	14.83061400	C	9.78225300	8.76442100	14.83167400
C	9.21664300	9.96391400	15.44463500	C	9.21580600	9.96448700	15.44525900
C	9.20186700	8.30031000	13.58862200	C	9.20075800	8.29990000	13.58913100
C	8.20554800	9.04772300	12.85166500	C	8.20476300	9.04698300	12.85235800
C	7.78353100	10.29352100	13.41360500	C	7.78218600	10.29198100	13.41531500
C	5.47572800	9.93424800	12.57204400	C	5.47508100	9.93292600	12.57231200
C	5.92686700	8.74368300	11.92766100	C	5.92677100	8.74280300	11.92775600
C	7.28589800	8.30033000	12.07143800	C	7.28553300	8.29971200	12.07184600

C	5.08877200	7.57696700	11.85232800	C	5.08869400	7.57628000	11.85270700
C	8.51053900	6.34881800	18.77745700	C	8.51105300	6.34985700	18.77770100
C	5.87659400	6.40885000	19.79185900	C	5.87683000	6.41027000	19.79149400
C	6.33211000	5.21532800	19.15972000	C	6.33281000	5.21782000	19.15781400
C	7.67760400	5.18118900	18.69948800	C	7.67844500	5.18203200	18.69929100
C	4.52089300	6.85284100	19.65140400	C	4.52068100	6.85377600	19.65134700
C	3.57838200	6.11681100	18.87989100	C	3.57798000	6.11842500	18.87914200
C	4.00111600	4.85694700	18.30411500	C	4.00184800	4.86038800	18.29980400
C	5.36313100	4.42089700	18.43672800	C	5.36414000	4.42725600	18.43095500
C	2.58103200	6.85661700	18.18542900	C	2.57993200	6.85716800	18.18599200
C	2.11124000	6.40517900	16.90269400	C	2.11146200	6.40466200	16.90260200
C	3.46112300	4.31507500	17.07057800	C	3.45857200	4.31242900	17.07066200
C	2.56537100	5.17695000	16.31183600	C	2.56289700	5.17466100	16.31243200
C	2.69517300	5.17102000	14.88529400	C	2.69309800	5.16747800	14.88522600
C	2.41767800	6.34889900	14.10128400	C	2.41937000	6.34783200	14.10322700
C	3.30022500	6.34983000	12.97288900	C	3.30207900	6.34923200	12.97533200
C	4.12803700	5.18191800	13.05040600	C	4.12849200	5.18109900	13.05205100
C	3.75605600	4.42188400	14.22978800	C	3.75348600	4.41648500	14.22895600
C	4.69291800	3.59642300	14.96436100	C	4.69036800	3.58800400	14.96272500
C	4.50840500	3.50111400	16.42304700	C	4.50690300	3.49796700	16.42221100
C	5.69884800	3.60640500	17.28860800	C	5.69886000	3.61249300	17.28442500
C	8.03356500	4.44170800	17.51753600	C	8.03489400	4.44224700	17.51751300
C	7.05350000	3.71186900	16.76870300	C	7.05421700	3.71553900	16.76719000
C	6.05245300	3.67962100	14.47199200	C	6.05191000	3.67900000	14.47233600
C	7.20972200	3.69823400	15.34805700	C	7.21068600	3.69837700	15.34719800
C	6.42434400	4.42396500	13.28043800	C	6.42458600	4.42412000	13.28087500
C	8.27736800	4.41297800	14.69092400	C	8.27827500	4.41201800	14.69001000
C	9.10113100	5.15437400	16.86448000	C	9.10164100	5.15495100	16.86412200
C	9.37550000	6.34571800	17.63839800	C	9.37517600	6.34707500	17.63796500
C	9.78051800	6.38869100	14.83229500	C	9.78155400	6.38825200	14.83188300
C	9.21507000	5.18802800	15.44578300	C	9.21563800	5.18792200	15.44538500
C	9.19976800	6.85311800	13.58954500	C	9.20049800	6.85269700	13.58923700
C	8.20464900	6.10576700	12.85221800	C	8.20466300	6.10555800	12.85240000
C	7.78155700	4.86119800	13.41578900	C	7.78201600	4.86061700	13.41550300
C	5.47457900	5.21958500	12.57145600	C	5.47501300	5.21963600	12.57229900
C	5.92682900	6.41006200	11.92688700	C	5.92676000	6.40971200	11.92769300
C	7.28560000	6.85317200	12.07115900	C	7.28551300	6.85280500	12.07181200
Dy	5.23461500	5.74308300	16.04995600	Dy	5.21232100	5.73840700	16.01622700

ωB97X-D/6-31G(d)~CEP-4G

**Spin multiplicity = 9**				**Spin multiplicity = 13**			
N	6.31953400	7.61353000	15.87465400	N	6.33951500	7.64015300	16.00152100

V	8.05483100	7.61940200	15.46523900	V	8.06006500	7.63932600	15.50684900
Dy	5.06849200	9.34645000	15.99398100	Dy	5.01666900	9.31455800	15.84997200
C	6.71112400	7.57266900	19.85862400	C	6.71323000	7.56818900	19.85905100
C	8.01154400	7.57261500	19.30568900	C	8.01418200	7.56900100	19.30746500
C	8.49508600	8.79211000	18.75588000	C	8.49715000	8.78863600	18.75664600
C	5.87783000	8.73346200	19.76841000	C	5.87994800	8.72787700	19.76605800
C	6.33152400	9.91781000	19.13581100	C	6.33306700	9.91130000	19.13134700
C	7.67044900	9.95661900	18.68070800	C	7.67186800	9.95200500	18.67826400
C	4.52457200	8.29460000	19.63382700	C	4.52675500	8.29076800	19.63440300
C	3.58493600	9.02946200	18.86924500	C	3.58849100	9.02491000	18.86682700
C	4.00114500	10.28874300	18.29480800	C	4.00781400	10.27644400	18.28838400
C	5.36371000	10.70309800	18.41465300	C	5.36788300	10.69095800	18.40892600
C	2.59492200	8.29438400	18.17647800	C	2.59609700	8.29346500	18.17472300
C	2.12658500	8.75173400	16.89573200	C	2.12602500	8.75247900	16.89446200
C	3.45111700	10.84415300	17.07515100	C	3.46045600	10.82647500	17.06872600
C	2.54929600	9.99091500	16.31487200	C	2.54317400	9.99304900	16.31290500
C	1.83728000	7.58076000	16.11214400	C	1.83304700	7.58047800	16.11225200
C	2.02030000	7.58016100	14.71309100	C	2.01778300	7.58200100	14.71399300
C	2.68800900	9.99577000	14.88731800	C	2.67054200	10.01408600	14.87960900
C	2.43420900	8.80752600	14.11615200	C	2.43589900	8.81344900	14.12037800
C	3.31790300	8.80234800	12.99642800	C	3.32305600	8.80486600	13.00303500
C	3.79740300	7.57810100	12.43727800	C	3.79628500	7.57958300	12.43624700
C	4.13585800	9.96813700	13.06765900	C	4.13251100	9.97666800	13.06590600
C	3.74952400	10.74191700	14.22747000	C	3.73124400	10.76670700	14.21421500
C	4.70143800	11.54185700	14.96551200	C	4.69752400	11.55347000	14.95725200
C	4.51449100	11.63050800	16.41892900	C	4.52387100	11.60518300	16.41023900
C	5.70069000	11.49525200	17.26539500	C	5.70613900	11.47042200	17.25630900
C	8.03027400	10.69489800	17.50496200	C	8.03248600	10.69121000	17.50410500
C	7.05527800	11.41247400	16.75664000	C	7.05658600	11.40306400	16.75317600
C	6.05602200	11.45456600	14.47895600	C	6.05481400	11.45911800	14.47484000
C	7.21395700	11.43860100	15.34722500	C	7.21349500	11.43883100	15.34365300
C	6.42916200	10.72010400	13.28951200	C	6.42977900	10.72587400	13.28304600
C	8.28066900	10.73743200	14.69153200	C	8.27952900	10.73968200	14.68875600
C	9.09035600	9.98650100	16.85144700	C	9.09139300	9.98462300	16.85045400
C	9.35821000	8.79105600	17.62187600	C	9.35955300	8.78735200	17.62180500
C	9.81697000	7.57134300	17.02996600	C	9.82304800	7.56801700	17.03249100
C	10.14571200	7.57604300	15.61659300	C	10.14726000	7.57616000	15.61743900
C	9.78069200	8.76405900	14.83304900	C	9.78016900	8.76515300	14.83333900
C	9.21939600	9.96886800	15.44123700	C	9.21860400	9.96951000	15.44009600
C	9.19098800	8.29953800	13.60272300	C	9.18246800	8.30065300	13.60792800
C	8.19750200	9.04535900	12.86814900	C	8.19412200	9.04716800	12.86842000
C	7.78196800	10.28825500	13.42090800	C	7.78201800	10.29276800	13.41647500
C	5.48071900	9.92813300	12.59000900	C	5.47973500	9.93383600	12.58555700
C	5.92923200	8.73944800	11.95693200	C	5.92619900	8.74303700	11.95503900

C	7.28119000	8.29906800	12.09726800	C	7.27792200	8.30145400	12.09799500
C	5.09443800	7.57792000	11.88094700	C	5.09149900	7.58056400	11.87778700
C	8.49746700	6.35192000	18.75757600	C	8.50040200	6.34791900	18.75932500
C	5.87593900	6.41206100	19.77075000	C	5.87723800	6.40706400	19.77375800
C	6.33064100	5.22357700	19.14536700	C	6.33131800	5.21831000	19.14930500
C	7.67066000	5.18774800	18.68674100	C	7.67173800	5.18463600	18.68790300
C	4.52420500	6.85791100	19.63106300	C	4.52537200	6.85507000	19.63298100
C	3.58572200	6.12628100	18.86576800	C	3.58729800	6.12607500	18.86532000
C	4.01244400	4.87164200	18.29213500	C	4.01703200	4.87314800	18.29151900
C	5.36452800	4.42400300	18.43162800	C	5.36524200	4.41433300	18.43804300
C	2.59096400	6.86309600	18.17689500	C	2.59120900	6.86281000	18.17601200
C	2.12021400	6.41480500	16.89976900	C	2.11690000	6.41564800	16.90018900
C	3.48043800	4.34126100	17.06135100	C	3.48941000	4.35265600	17.05598500
C	2.58340700	5.19960300	16.30979800	C	2.58848800	5.20736000	16.30685600
C	2.71025900	5.18954800	14.89165000	C	2.71098600	5.19712300	14.89020700
C	2.43045000	6.35646700	14.10964600	C	2.42609600	6.36062100	14.10666400
C	3.31129500	6.35522300	12.98658100	C	3.30646300	6.35824800	12.98233900
C	4.13435000	5.19334300	13.06349700	C	4.13149400	5.19761700	13.06112800
C	3.76669500	4.43916300	14.24078400	C	3.76856800	4.45000900	14.24217600
C	4.69557300	3.61148100	14.96831800	C	4.69706900	3.62558300	14.96976500
C	4.51055900	3.51190500	16.42348000	C	4.51288800	3.51843200	16.42239500
C	5.69795100	3.60482300	17.29130500	C	5.69875800	3.59104900	17.29752500
C	8.02624600	4.44779300	17.50962100	C	8.02517700	4.44472000	17.50977400
C	7.05252900	3.70897400	16.76973000	C	7.05253700	3.69803300	16.77242500
C	6.05172500	3.68133500	14.47540700	C	6.05145700	3.68093600	14.47365800
C	7.20586000	3.70162800	15.35253800	C	7.20405700	3.69734700	15.35253600
C	6.42256700	4.43241100	13.29377000	C	6.41940500	4.43504000	13.29488600
C	8.26954500	4.42017100	14.69961500	C	8.26644200	4.42145000	14.70115200
C	9.09053800	5.15781100	16.85716400	C	9.09143300	5.15470600	16.85863300
C	9.36445700	6.34889400	17.62747400	C	9.36878400	6.34436600	17.63022100
C	9.75650800	6.39408400	14.84140500	C	9.74677000	6.39689400	14.84524400
C	9.20999900	5.18390100	15.44813800	C	9.20694100	5.18379000	15.44980600
C	9.18051200	6.85684500	13.60781200	C	9.16806100	6.86030400	13.61540000
C	8.19146300	6.11060100	12.87191900	C	8.18563400	6.11398400	12.87498200
C	7.77280200	4.86979000	13.43019500	C	7.76847100	4.87366400	13.43326700
C	5.47644300	5.22858400	12.58966000	C	5.47304000	5.23178200	12.58943500
C	5.92744200	6.41581800	11.95497100	C	5.92448700	6.41815600	11.95316500
C	7.27971500	6.85571700	12.09755900	C	7.27585900	6.85840800	12.09846100
Dy	5.34111800	5.71703000	16.05338500	Dy	5.40627500	5.71312300	16.09472900

S8. Reference

S1. Yang, S. *et al*. Mixed metal nitride clusterfullerenes in cage isomers: $Lu_xSc_{3-x}N@C_{80}$(x=1, 2) as compared with $M_xSc_{3-x}N@C_{80}$(M =Er, Dy, Gd, Nd). *J. Phys. Chem. C* **113**, 7616–7623 (2009).

S2. Yang, S. & Dunsch, L. A large family of dysprosium-based trimetallic nitride endohedral fullerenes: $Dy_3N@C_{2n}$ (39≤n≤44). *J. Phys. Chem. B* **109**, 12320-12328(2005)

S3. Wei, T. *et al.* Entrapping a group-VB transition metal, vanadium, within an endohedral metallofullerene: $V_xSc_{3-x}N@I_h$-C_{80}(x =1,2). *J. Am. Chem. Soc.* **138**, 207–214 (2016).

S4. Wei, T. *et al.* An expanded family of dysprosium-scandium mixed-metal nitride clusterfullerenes: the role of the lanthanide metal on the carbon cage size distribution. *Chem. Eur. J.* **21**, 5750–5759 (2015).

S5. Wei, T. *et al.* Blending non-group-3 transition metal and rare-earth metal into a C_{80} fullerene cage with D_{5h} symmetry. *Angew. Chem. Int. Ed.* **57**, 10273–10277 (2018).

S6. Krylov, D. S. *et al.* Magnetization relaxation in the single-ion magnet $DySc_2N@C_{80}$: quantum tunneling, magnetic dilution, and unconventional temperature dependence. *Phys. Chem. Chem. Phys.* **20**, 11656–11672 (2018).

S7. Koponen, I. Random transition rate model of stretched exponential relaxation. *J. Non-Cryst. Solids* **189**, 154–160 (1995).

S8. Wang, J. *et al.* Opening magnetic hysteresis by axial ferromagnetic coupling: from mono-decker to double-decker metallacrown. *Angew. Chem. Int. Ed.* **60**, 5299–5306 (2021).

S9. Langley, S. K. *et al.* A $\{Cr^{III}{}_2Dy^{III}{}_2\}$ single-molecule magnet: enhancing the blocking temperature through 3d magnetic exchange. *Angew. Chem. Int. Ed.* **52**, 12014–12019 (2013).

S10. Akhtar, M. N. *et al.* Exploring the role of intramolecular interactions in the suppression of quantum tunneling of the magnetization in a 3d-4f single-molecule magnet. *Inorg. Chem.* **60**, 9302–9308 (2021).

S11. Mondal, K. C. *et al.* Coexistence of distinct single-ion and exchange-based mechanisms for blocking of magnetization in a $Co^{II}{}_2Dy^{III}{}_2$ single-molecule magnet. *Angew. Chem. Int. Ed.* **51**, 7550–7554 (2012).

S12. Liu, J.-L. *et al.* A heterometallic Fe^{II}-Dy^{III} single-molecule magnet with a record anisotropy barrier. *Angew. Chem. Int. Ed.* **53**, 12966–12970 (2014).

S13. Li, J. *et al.* Tuning quantum tunnelling of magnetization through 3d–4f magnetic interactions: an alternative approach for manipulating single-molecule magnetism. *Inorg. Chem. Front.* **4**, 114–122 (2017).

S14. Kotrle, K. *et al.* 3d – 4f magnetic exchange interactions and anisotropy in a series of heterobimetallic vanadium(IV)–lanthanide(III) Schiff base complexes. *Dalton Trans.* **50**, 13883–13893 (2021).

S15. Chibotaru, L. F. & Ungur, L. *Ab initio* calculation of anisotropic magnetic properties of complexes. I. Unique definition of pseudospin Hamiltonians and their derivation. *J. Chem. Phys.* **137**, 064112 (2012).

S16. Chibotaru, L. F. *et al.* Structure, magnetism, and theoretical study of a mixed-valence $Co^{II}_3Co^{III}_4$ heptanuclear wheel: lack of SMM behavior despite negative magnetic anisotropy. *J. Am. Chem. Soc.* **130**, 12445–12455 (2008).

S17. Chibotaru, L. F., Ungur, L. & Soncini, A. The origin of nonmagnetic Kramers doublets in the ground state of dysprosium triangles: evidence for a toroidal magnetic moment. *Angew. Chem. Int. Ed.* **47**, 4126–4129 (2008).

S18. Aquilante, F. *et al.* MOLCAS 8: New capabilities for multiconfigurational quantum chemical calculations across the periodic table. *J. Comput. Chem.* **37**, 506–541 (2016).

S19. Roos, B. O. *et al.* New relativistic ANO basis sets for actinide atoms. *Chem. Phys. Lett.* **409**, 295–299 (2005).

S20. Roos, B. O. *et al.* Main group atoms and dimers studied with a new relativistic ANO basis set. *J. Phys. Chem. A* **108**, 2851–2858 (2004).

S21. Finley, J. *et al.* The multi-state CASPT2 method. *Chem. Phys. Lett.* **288**, 299–306 (1998).

S22. Frisch, M. J. *et al.* Gaussian, Inc., Wallingford CT, (2016).

S23. Glendening, E.D. *et al.* Version 3.1, University of Wisconsin, Madison, WI, (1996).
